# Human milk fatty acid composition and its association with maternal blood and adipose tissue fatty acid content in a cohort of women from Europe

**DOI:** 10.1007/s00394-021-02788-6

**Published:** 2022-01-24

**Authors:** Francesca Giuffrida, Mathilde Fleith, Amélie Goyer, Tinu Mary Samuel, Isabelle Elmelegy-Masserey, Patric Fontannaz, Cristina Cruz-Hernandez, Sagar K. Thakkar, Cathriona Monnard, Carlos Antonio De Castro, Luca Lavalle, Thameur Rakza, Massimo Agosti, Isam Al-Jashi, Almerinda Barroso Pereira, Maria Jose Costeira, Giovanna Marchini, Mireille Vanpee, Tom Stiris, Sylvia Stoicescu, Maria Gorett Silva, Jean-Charles Picaud, Cecilia Martinez-Costa, Magnus Domellöf, Claude Billeaud

**Affiliations:** 1 Nestlé Research, Vers-chez les-Blanc, 1000 Lausanne 26, Switzerland; 2 Nestlé Research, 29 Quality Road, Singapore, 618802 Singapore; 3 Centre d’Investigation Clinique de Lille, Hôpital Jeanne de Flandre, 59777 Lille, France; 4 Ospedale del Ponte, Varese, Italy; 5 Private Med. Practice, Bucharest, Romania; 6 Hospital de São Marcos, 4700-327 Braga, Portugal; 7 ICVS (Population Health Assessment)-Minho Medical School, Braga, Portugal; 8 Karolinska University Hospital, Stockholm, Sweden; 9 Oslo University Hospital, Oslo, Norway; 10 Polizu Hospital, Bucharest, Romania; 11 Hospital de S. João, 4200-319 Porto, Portugal; 12 Hospices Civils de Lyon, Neonatology, Hôpital de La Croix Rousse, Hospices civils de Lyon, 69004 Lyon, France; 13 Univ. Lyon, Carmen Laboratory, INSERM, INRA, Université Claude Bernard Lyon 1, 69921 Oullins, France; 14 Hospital Clínico Universitario, University of Valencia, Valencia, Spain; 15 Department of Clinical Sciences/Pediatrics, Umeå University, Umeå, Sweden; 16 Neonatology & Nutrition, CIC Pédiatrique 1401 Inserm, CHU de Bordeaux, Bordeaux, France; 17 Nestlé Product Technology Center-Nutrition, Société des Produits Nestlé S.A., 1800 Vevey, Switzerland

**Keywords:** Fatty acids, Human milk, Nutrition, Lipids, Plasma, Erythrocytes, Pregnancy, Lactation, Adipose tissue, DHA, LCPUFA

## Abstract

**Purpose:**

Human milk (HM) composition is influenced by factors, like maternal diet and body stores, among other factors. For evaluating the influence of maternal fatty acid (FA) status on milk FA composition, the correlation between FA content in HM and in maternal plasma, erythrocytes, and adipose tissue was investigated.

**Methods:**

223 European women who delivered at term, provided HM samples over first four months of lactation. Venous blood and adipose tissue (only from mothers who consented and underwent a C-section delivery) were sampled at delivery. FAs were assessed in plasma, erythrocytes, adipose tissue, and HM. Evolution of HM FAs over lactation and correlations between FA content in milk and tissues and between mother’s blood and cord blood were established.

**Results:**

During lactation, arachidonic acid (ARA) and docosahexaenoic acid (DHA) significantly decreased, while linoleic acid (LA), alpha-linolenic acid (ALA), and eicosapentaenoic acid (EPA) remained stable. Positive correlations were observed between HM and adipose tissue for palmitic, stearic, oleic, and polyunsaturated fatty acids (PUFAs). Correlations were found between milk and plasma for oleic, LA, ARA, ALA, DHA, monounsaturated fatty acids (MUFAs), and PUFAs. No correlation was observed between erythrocytes and HM FAs. LA and ALA were more concentrated in maternal blood than in infant blood, contrary to ARA and DHA, supporting that biomagnification of LCPUFAs may have occurred during pregnancy.

**Conclusions:**

These data show that maternal adipose tissue rather than erythrocytes may serve as reservoir of PUFAs and LCPUFAs for human milk. Plasma also supplies PUFAs and LCPUFAs to maternal milk. If both, adipose tissue and plasma PUFAs, are reflection of dietary intake, it is necessary to provide PUFAs and LCPUFAs during pregnancy or even before conception and lactation to ensure availability for mothers and enough supply for the infant via HM.

**Supplementary Information:**

The online version contains supplementary material available at 10.1007/s00394-021-02788-6.

## Introduction

Human milk (HM) is the ideal food in early infant life. Lipid content of HM is reported to vary with progression of lactation ranging from 1.9 to 2.3% in colostrum to 3.2–4.9% in mature milk and represents approximately half of energy provided to the infant when exclusively breastfed [1–3].

Among milk lipids, triacylglycerols is the most abundant class representing over 90% of total lipids. Triacylglycerols consist of a molecule of glycerol esterified with three molecules of fatty acids (FAs). FAs in HM can be (1) generated by de novo fatty acid synthesis in the mammary gland, (2) mobilized from maternal stores, i.e., adipose tissue, and (3) directly supplied by the maternal intake (diet and supplements) [4, 5]. The presence in the human mammary gland cell of an enzyme allowing termination of de novo FA synthesis at 8, 10, 12, and 14 carbons explains the presence of medium and long-chain saturated fatty acids (SFAs) in HM [5, 6]. Among polyunsaturated fatty acids (PUFAs), linoleic acid (LA; 18:2 n-6) and alpha-linolenic acid (ALA; 18:3 n-3) are essential FAs because they cannot be synthetized in humans. Stable isotope studies showed that 30% of HM LA and 65% of HM ALA are directly transferred from the diet, while the other part is derived from body stores [7–9]. LA and ALA are precursors of n-6 and n-3 long-chain PUFAs (LCPUFAs), like arachidonic (ARA; 20:4 n-6), eicosapentaenoic acid (EPA; 20:5 n-3), and docosahexaenoic acid (DHA; 22:6 n-3), which are involved in inflammatory reactions and brain functions and growth [10]. Since the conversion of LA to ARA and of ALA to EPA and DHA is < 1% [7, 11], HM LCPUFAs are mainly derived from diet, either directly or, in higher proportions, after incorporation and further mobilization from body stores. A review, including 78 studies from thirty-three European, seven African, five Eastern Mediterranean, fourteen Asian, seventeen North American, five South American, and five Australian countries [12], showed that DHA level in HM ranged between 0.1% in Sudan and 0.84% in Malaysia and variations are attributable mostly to dietary habits, even if socio-economic factor and also genetic polymorphisms may play a role.

There is lack of comprehensive research on maternal and term HM FA status across European countries. The largest best designed cross-country study with detailed FA profiles of HM (9 countries) did not include European countries other than UK and only included one time point of assessment [13]. Besides studies in individual countries, e.g., Spain [14], Italy [15], France [16], or Greece [17], which were based on small sample sizes, there is no study evaluating the FA status of mothers and babies in Europe.

Foremilk and hindmilk are known to differ in lipid concentration [18, 19] and PUFA concentration [20] but only few data on the composition of HM FA on full expressed breast milk are available, i.e., from women living in Asia [21, 22].

Several studies assessed the relationship between HM FAs and FA status in blood (plasma or erythrocytes) [23, 24] or adipose tissue [25] in mothers and newborn infants, showing that maternal FA status (blood, adipose tissue) affects HM FA composition. Comparison of the maternal and neonate FA status at birth showed a higher absolute concentration of all plasma lipids in maternal blood than in cord blood (neonate), while the proportions of DHA and ARA were higher in the neonate [26]. This potential preferential transfer of DHA and ARA from the mother to the infant has been named “biomagnification” [27] and seems to be highly variable at the individual level.

The present study aimed at

- assessing the FA profile of colostrum, transitional, and mature HM over first four months of lactation, in a large number of mothers having delivered term infants in European countries;
- evaluating the correlation between the maternal FA status (plasma, erythrocyte, adipose tissue) and milk FA composition of lipidome. The correlation between plasma, erythrocytes, and adipose tissue LA and milk ARA was also investigated, LA being the precursor of ARA. Similarly, the correlations between plasma and erythrocytes ALA and milk EPA and DHA were investigated, ALA being the precursor of both EPA and DHA. To evaluate the correlation between the maternal FA status and milk FA composition, lipid classes, e.g., phospholipids, triacyglycerols etc., were not separated and fatty acid profile of whole lipidome was measured in order to assess which tissues among plasma, erythrocyte and adipose tissue influences FA human milk composition.
- evaluating the correlation between the maternal blood FAs and infant blood FAs at birth;
- evaluating factors influencing FA composition of HM.

## Materials and methods

### Study design and population

The data presented are part of an observational, longitudinal, multicentric study to characterize the HM in the first four months of lactation (Atlas of Human Milk Nutrients study). This study was performed in 13 centers in Europe, including Spain, France (3 centers), Italy, Norway, Portugal (3 centers), Romania (2 centers), and Sweden (2 centers). Institutional and ethical board of each center approved this study (France: ‘comité de protection des personnes sud-ouest et outre mer III’; Italy: Comitato Etico Ospedale di Circolo Fondazione Macchi; Norway: Regionale Komiteer for Medisinsk og Helsefaglig Forskningetikk; Portugal: Comissao de Etica do Centro Hospitalar Sao Joao, Comissao de Etica para a Saude do Hospital de Braga, Conselho de Administracao do Centro Hospitalar de Alto Ave; Romania: Comisia Locala de Etica, Spitalul Clinic Judetean de Urgenta Ilfov, Ethic Committee of Institutul Pentru Ocrotirea Namei si Copilului; Spain: Comité Ético de Investigación Clínica Del Hospital Clínico Universitario de Valencia; Sweden: Regionala etikprövningsnämnden Umeå). Written informed consent was obtained from all participants after they received explanations in their respective local languages and have read and understood the purpose and the objectives of this study. This study was registered at www.ClinicalTrials.gov (NCT01894893).

Pregnant women were recruited before delivery, generally during the last trimester of pregnancy. The inclusion criteria for enrollment in this study were as follows: (1) age from 18 to 40 years; (2) BMI before pregnancy between 19 and 29, willing to predominantly breastfeed their newborn baby at least until 4 months of age. Administration of infant formula or donor milk complementing mother milk along study participation was accepted only if properly documented and justified and if occasional and not lasting more than 7 consecutive days; (3) permanently residing at a maximum distance of 50 km from the hospital/center of investigation for the duration of this study; and (4) agreeing to the study protocol and signing informed consent form. The exclusion criteria were as follows: (1) currently participating in another trial; (2) presenting any disease or medical condition which prevent breastfeeding or collection of human milk samples or for which breastfeeding is not indicated; (3) medical conditions (e.g., gestational diabetes) or on medications for conditions such as metabolic and cardiovascular abnormalities; (4) dietary problems, such as anorexia or bulimia; and (5) subjects not able to comply to the study procedures. Dedicated, trained, and certified research nurses and assistants collected all data for this study. Maternal data on socio-demographic characteristics, age, body weight, height, mode of delivery, medical history, and history of dietary supplements were collected by means of an interview-administrated questionnaire and consulting medical records. Three-day food diaries were filled, and the results will be discussed in a separated manuscript. Gestational age was calculated based on the last menstrual period and confirmed by ultrasound. Newborn characteristics included demography (gender and unique child), anthropometry, history of medication use, body composition (one center in France and one in Sweden), and infant HM intake diary (three centers in France only). Singleton pregnancy was not criteria for recruitment.

### Assessment of maternal factors

Self-reported maternal pre-pregnancy weight and height was used to calculate the pre-pregnancy BMI (ppBMI). The ppBMI was calculated as weight (in kilograms) divided by height (in meters) squared (kg/m^2^). The ppBMI was categorized based on the WHO guidelines [28] into normal weight (NW: 18.5–24.9 kg/m^2^) and overweight (OW: 25.0–29.9 kg/m^2^). No underweight women were identified in this study. For mode of delivery we categorized mothers as those giving birth via Cesarean section (C-section) versus vaginal delivery. Information on emergency or elective C-section was not collected.

**Fig. 1:**
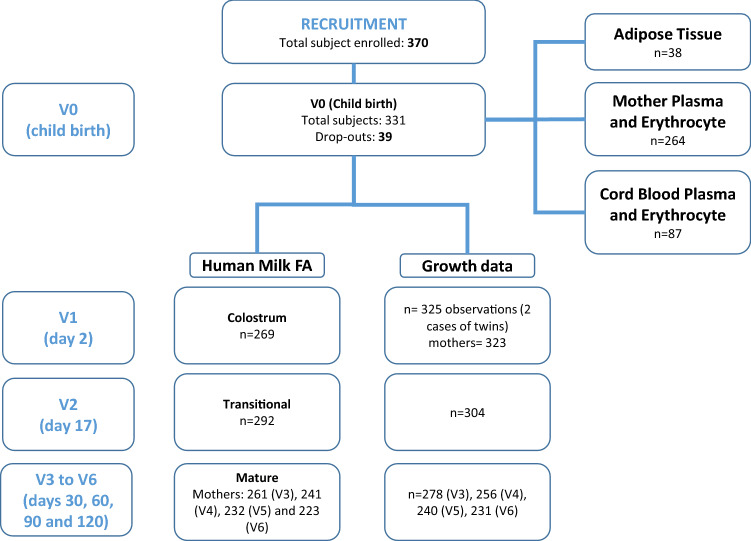
Study flow chart for subject recruitment and sampling

Figure 1 shows the flow chart of subject recruitment and data capture of sampling.

### Human milk collection

Human milk samples of all enrolled women were collected from the same breast in the morning, at the same time (at 11h00 ± 2 h) using an electric breast pump (Medela Symphony, Switzerland). In order to obtain full expressed milk, it was asked to empty the breast in the previous feed. These collected single full breast milk samples were mixed, and an aliquot of 5–10 mL for the first time point and 10–40 mL HM for all other time points were collected. The remainder of the HM was kept by the mother for future use. Each collected HM sample was transferred to freezing tubes, labeled with subject number and collection information, stored at − 80 °C, and then shipped on dry ice to the Nestlé Research Centre (Lausanne, Switzerland) for storage at − 80 °C until analysis. The frozen HM samples were thawed once for aliquoting into 15 individual small volume fractions (0.2–2 mL) in separate polypropylene tubes dedicated to the different analyses. For FA analysis, for each sample, 250 µL aliquots were transferred in labeled tubes and stored at − 80 °C until analysis. Milk was collected at the following time points: 0–3 days after birth (visit V1), day 17 ± 3 (visit V2), day 30 ± 3 (visit V3), day 60 ± 5 (visit V4), day 90 ± 5 (visit V5), and day 120 ± 5 (visit V6).

### Plasma and erythrocytes collection and preparation

Blood was collected from mothers either overnight or 12 h of fasting, median cubital vein by a phlebotomist or a trained nurse within 72 h after delivery. Approximately 9 mL of blood was collected in tubes containing EDTA as anticoagulants for FA analysis. Umbilical cord blood was recovered after delivery in a sub-set of infants (France, Portugal, Romania, and Sweden gave consent) for measuring FA status of infants following the same procedure.

Blood samples were immediately treated, and plasma and erythrocytes were prepared for FA analysis. Briefly, after collection, tubes were gently inverted to mix without causing hemolysis before processing and centrifuged at 626×*g* for 10 min. Blood tubes were kept on ice and erythrocytes and plasma were sampled as 200 µL aliquots and added into a 10 mL screw cap glass test tube containing 200 µL of ethanol for plasma and 200 µL of a lysis buffer for erythrocytes as described by Cruz-Hernandez et al. [29, 30]. Buffy coat was discarded before sampling the erythrocytes. The tubes were equipped with Teflon-lined screw caps perfectly fitting the test tubes. Tubes were vortexed for 10 s before storage at − 80 °C until lipid analysis.

### Adipose tissue collection

Subcutaneous adipose tissue corresponding to 1 cm^3^ was collected from a sub-set of mothers undergoing elective Caesarian section. The tissue was transferred in a tube and stored at − 80 °C until analysis.

### Fatty acid and total fat analysis

HM, erythrocytes, and plasma lipid FA were quantified as described by Cruz-Hernandez et al. [29, 30]. Adipose tissues were homogenized by CryPre CP02 (LGC Genomics Limited, Hoddesdon, UK). After addition of 200 µL of internal standard FA methyl ester (FAME) 23:0 (17 mg/mL) and 50 µL of internal standard triglyceride 11:0 (5 mg/mL), followed by 2 mL of methanol, 2 mL of methanol/HCL (3 N), and 2 mL of n-hexane, the tubes were heated at 100 °C for 60 min. After cooling down to room temperature, 2 mL of water was added. After centrifugation at 1200×*g* for 5 min the upper phase (hexane) was injected into a gas chromatograph coupled with flame ionization detector.

Analysis of erythrocytes, plasma, and adipose tissue FA was performed with a similar method as previously described for different fat matrices [31].

Total fat was measured by mid-infrared human milk analyzer (MIRIS AB, Uppsala, Sweden) as described by Giuffrida et al. [32].

### Statistics

The data were not imputed and classified in the following manner when it comes to lactation stage: all observations at visit 1 were considered colostrum (≤ 5 days postpartum), observations at visit 2 (between 14 and 20 days postpartum) were considered transitional milk, and observations beyond 20 days were considered mature milk.

All statistical analyses were performed with the statistical software R version 3.2.3 (R, 2019) [33]. The levels of FAs in each time point were compared to the levels of the subsequent time point (*e.g.,* V1 vs V2, V2 vs V3, etc.). Relative FAs levels were considered, i.e., levels in g were divided into 100 g of total fatty acids. A linear mixed model was used considering the within subject variability and comparing the fixed effect of child’s age (according to visit). Diagnostic checks (normality of residuals, homoscedasticity, etc.) for linear mixed effect models were run. Spearman correlations were run between HM at different lactation stages (colostrum, transitional milk, and mature milk (average of visit 3 to visit 6)) and mother plasma, erythrocyte, and adipose tissue at delivery, for a sub-set of FAs. Correlations were calculated with cor.test function and plots were done with the ggplot2 library. When absolute values of rho ≥ 0.4 the correlation was considered as strong and when p < 0.05 it was in addition considered as significant. Due to low number of observations, it was not possible to perform comparison among HM FA profile from different countries.

## Results

### Study population

The flow chart of subject recruitment and data capture of sampling is shown in Fig. 1.

Of the 370 women originally enrolled, 223 mothers completed the study providing at least one HM sample. 39 dropped out at childbirth (V0) and 8 within 1–3 days after delivery (V1); thus, growth data were obtained from 323 mothers at V1. Colostrum FAs were obtained from 269 participants (V1) and human milk FAs from 292 (V2), 261 (V3), 241 (V4), 232 (V5), and 223 (V6) participants. Reasons and numbers for dropouts can be found elsewhere [34].

The characteristics of the 293 mothers and their newborns are summarized in Table 1. The 293 participants were from Spain (4.1%), France (29.0%), Italy (3.1%), Norway (3.4%), Portugal (32.8%), Romania (14.3%), and Sweden (13.3%). Mean age at recruitment was 31.2 years (standard deviation, SD: 4.3). The newborn population showed a predominance of male (54.6%) and firstborn children (66.9%). Most mothers had vaginal delivery (74.1%) and 25.9% gave birth by Cesarean section (C-section). Mean gestational age was 39.3 weeks (SD: 1.2). Mean pre-pregnancy maternal weight was 61.71 kg (SD: 8.11), and based on ppBMI, 229 women were NW (79%) and 61 women were OW (21%). The mean (SD) height and ppBMI were 164.82 (6.13) and 22.71 (2.67), respectively. Most mothers were in the medium post-pregnancy weight loss category.

**Table 1:** Characteristics of lactating mothers and their infants having FA data on at least one point

	Unit	Level	Number of participants
*n* of mothers at V1 (day 2)			**323**
*n* of subjects having FA data on at least one point			**293**

	Unit	Level	Mean (SD)
Country *n* (%)		Spain	12 (4.1)
		France	85 (29.0)
		Italy	9 (3.1)
		Norway	10 (3.4)
		Portugal	96 (32.8)
		Romania	42 (14.3)
		Sweden	39 (13.3)
Mean age of mother (SD)	years		31.2 (4.3)
Infant Gender *n* (%)		F	133 (45.4)
		M	160 (54.6)
Mean parity (SD)			1.34 (0.6)
Unique child (%)		Unique	216 (66.9)
		With/siblings	107 (33.1)
Mode of delivery *n* (%)		Caesarean	76 (25.9)
		Vaginal	217 (74.1)
Mean infant gestational age (SD)	weeks		39.3 (1.2)
Mean pre-pregnancy weight (SD)	kg		61.71 (8.11)
Mean pre-pregnancy height (SD)	cm		164.82 (6.13)
Mean pre-pregnancy BMI (SD)	kg/m^2^		22.71 (2.67)
Mean infant body weight at birth (SD)	kg		3.35 (0.47)
Mean infant body length at birth (SD)	cm		49.91 (2.14)
Mean infant head circumference at birth (SD)	cm		34.47 (1.48)

Data are expressed as mean and standard deviation (SD)

*BMI* body max index

^1^Pre-pregnancy BMI (ppBMI) was categorized based on the WHO guidelines into normal (NW: 18.5–24.9 kg/m^2^) and overweight (OW: 25.0–29.9 kg/m^2^)

### Milk fatty acids

When presenting the results in the subsequent sections, all FA levels were summarized by mean ± SD since most of the relative FA levels approximated the normal distribution.

**Fig.2:**
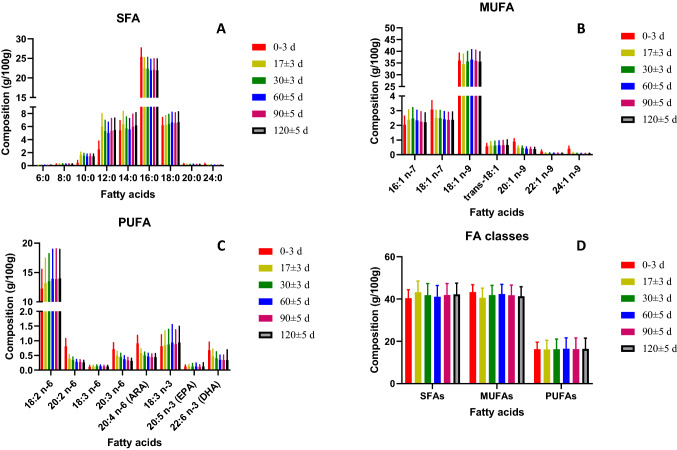
**A** SFAs (saturated fatty acids); **B** MUFAs (monounsaturated fatty acids); **C** PUFAs (polyunsaturated fatty acids) composition over lactation; **D** total SFAs, MUFAs, and PUFAs over lactation. Bars represent standard deviation

Fatty acid profiles of lipids in HM were obtained in colostrum (days 0–3), transitional (day 17), and mature (days 30, 60, 90, and 120) milk. Table and Fig. 2 show the FA composition of HM (g/100 g of total FAs) (mean ± standard deviation (SD)) at the different time points and the number of observations for each of the FA. For example, for caproic acid (6:0), at 0–3 days after delivery, 269 samples were analyzed, but this FA was detected in only two samples. For each FA, Table 2 also shows if the difference observed between visits are statistically significant, thus if there is an evolution over time of lactation. The milk FA composition expressed as mg FA/100 mL HM is given in supplementary Table 1. Supplementary Tables 2 and 3 list FA contents (mean ± standard deviation (SD)) of colostrum, transitional, and mature milk in g FA/100 g of total FAs and in mg/100 mL milk, in the different countries.

**Table 2:** Human milk fatty acid composition (g/100 g of total FA) at different lactation stages (mean ± standard deviation) and number of observations

Sampling days	0–3 days	17 ± 3 days	30 ± 3 days	60 ± 5 days	90 ± 5 days	120 ± 5 days
	*N* = 269	*N* = 292	*N* = 261	*N* = 241	*N* = 232	*N* = 223
SFAs	40.44 ± 4.00	43.20 ± 5.34*	41.82 ± 5.50*	41.09 ± 5.32	41.93 ± 5.40	42.23 ± 5.29
6:0	0.06 ± 0.00 (2)	0.06 ± 0.02 (26)	0.06 ± 0.01 (53)	0.07 ± 0.02 (88)	0.07 ± 0.01 (101)	0.08 ± 0.02 (107)
8:0	0.17 ± 0.09 (22)	0.22 ± 0.06 (253)	0.22 ± 0.06 (225)	0.21 ± 0.06 (216)	0.22 ± 0.06 (209)	0.22 ± 0.06 (191)
10:0	0.45 ± 0.34 (232)	1.63 ± 0.42* (292)	1.53 ± 0.34 (225)	1.44 ± 0.33 (241)	1.45 ± 0.35 (232)	1.44 ± 0.34 (223)
12:0	2.50 ± 1.29 (232)	5.97 ± 2.01* (292)	5.30 ± 1.65* (225)	5.02 ± 1.64 (241)	5.34 ± 1.88 (232)	5.46 ± 1.84 (223)
14:0	5.40 ± 1.51 (232)	6.35 ± 1.97* (292)	5.71 ± 1.76* (225)	5.55 ± 1.66 (241)	5.98 ± 1.93 (232)	6.19 ± 1.93 (223)
16:0	25.39 ± 2.27 (269)	22.51 ± 2.67* (292)	22.39 ± 2.86 (261)	21.94 ± 2.82* (241)	22.10 ± 2.81 (232)	21.94 ± 2.92 (223)
18:0	6.20 ± 1.19 (232)	6.22 ± 1.45 (292)	6.39 ± 1.48 (225)	6.64 ± 1.56 (241)	6.55 ± 1.56 (232)	6.68 ± 1.59 (223)
20:0	0.24 ± 0.09 (246)	0.18 ± 0.05* (277)	0.18 ± 0.03 (243)	0.18 ± 0.04 (221)	0.17 ± 0.04* (207)	0.17 ± 0.04 (194)
24:0	0.25 ± 0.12 (251)	0.10 ± 0.03* (203)	0.08 ± 0.02* (148)	0.07 ± 0.02 (102)	0.07 ± 0.02 (81)	0.07 ± 0.03 (87)
MUFAs	43.29 ± 3.47	40.63 ± 4.56*	41.90 ± 4.58*	42.38 ± 4.54	41.76 ± 4.85	41.34 ± 4.48
16:1 n-7	2.04 ± 0.59 (232)	2.38 ± 0.68* (292)	2.47 ± 0.73 (225)	2.34 ± 0.68 (241)	2.25 ± 0.67 (232)	2.21 ± 0.64* (223)
18:1 n-7	3.08 ± 0.60 (232)	2.51 ± 0.51* (292)	2.48 ± 0.54 (225)	2.41 ± 0.53 (241)	2.36 ± 0.53 (232)	2.38 ± 0.53 (223)
18:1 n-9	36.08 ± 3.14 (269)	34.45 ± 4.29* (292)	35.72 ± 4.25* (261)	36.46 ± 4.25 (241)	35.98 ± 4.48 (232)	35.59 ± 4.17 (223)
*trans*-18:1	0.57 ± 0.19 (261)	0.62 ± 0.27 (292)	0.62 ± 0.30 (261)	0.64 ± 0.30 (240)	0.65 ± 0.32 (231)	0.66 ± 0.35 (222)
20:1 n-9	0.89 ± 0.20 (261)	0.47 ± 0.13* (292)	0.45 ± 0.13* (261)	0.41 ± 0.11* (240)	0.39 ± 0.12* (228)	0.38 ± 0.12 (217)
22:1 n-9	0.22 ± 0.10 (236)	0.10 ± 0.03* (197)	0.09 ± 0.04* (157)	0.08 ± 0.03* (108)	0.08 ± 0.03 (91)	0.08 ± 0.03 (93)
24:1 n-9	0.41 ± 0.17 (266)	0.11 ± 0.05* (215)	0.08 ± 0.02* (147)	0.07 ± 0.02* (89)	0.07 ± 0.01 (74)	0.08 ± 0.02 (66)
PUFAs	16.26 ± 3.37	16.16 ± 4.37	16.27 ± 4.81	16.53 ± 5.14	16.30 ± 5.30	16.43 ± 5.07
18:2 n-6	12.26 ± 3.26 (269)	13.21 ± 4.22* (292)	13.53 ± 4.67 (261)	13.94 ± 5.02 (241)	13.86 ± 5.18 (232)	14.00 ± 4.95 (223)
20:2 n-6	0.81 ± 0.26 (266)	0.39 ± 0.13* (292)	0.34 ± 0.10* (260)	0.28 ± 0.08* (240)	0.27 ± 0.08* (221)	0.26 ± 0.07 (212)
18:3 n-6	0.11 ± 0.05 (20)	0.12 ± 0.05 (197)	0.13 ± 0.05(195)	0.13 ± 0.04 (180)	0.12 ± 0.04 (163)	0.12 ± 0.04 (144)
20:3 n-6	0.71 ± 0.22 (267)	0.48 ± 0.16* (292)	0.44 ± 0.13* (260)	0.37 ± 0.12* (241)	0.33 ± 0.10* (225)	0.31 ± 0.09 (216)
20:4 n-6 (ARA)	0.91 ± 0.27 (269)	0.58 ± 0.14* (292)	0.50 ± 0.11* (261)	0.46 ± 0.11* (241)	0.44 ± 0.11* (231)	0.44 ± 0.12 (219)
18:3 n-3	0.81 ± 0.39 (264)	0.85 ± 0.48* (269)	0.87 ± 0.52 (261)	0.94 ± 0.61 (241)	0.88 ± 0.49 (232)	0.94 ± 0.55 (221)
20:5 n-3 (EPA)	0.12 ± 0.05 (44)	0.12 ± 0.07 (131)	0.12 ± 0.10* (121)	0.12 ± 0.07 (103)	0.11 ± 0.07 (102)	0.12 ± 0.13 (87)
22:6 n-3 (DHA)	0.68 ± 0.27 (266)	0.47 ± 0.25* (291)	0.40 ± 0.22* (257)	0.35 ± 0.16* (233)	0.34 ± 0.17 (222)	0.34 ± 0.35 (212)
Ratios and sums						
ARA/DHA	1.52 ± 0.76 (269)	1.47 ± 0.75 (291)	1.52 ± 0.75 (261)	1.62 ± 0.77 (241)	1.59 ± 0.81* (232)	1.68 ± 0.89 (223)
EPA + DHA	1.03 ± 0.34 (44)	0.71 ± 0.35 (131)	0.62 ± 0.33 (121)	0.54 ± 0.25 (131)	0.53 ± 0.24 (102)	0.57 ± 0.64 (87)
Total fat (g/100 mL)	2.7 ± 1.0	4.0 ± 1.3	4.0 ± 1.5	4.1 ± 1.5	4.0 ± 1.7	4.1 ± 1.8

*SFA* saturated fatty acid, *MUFA* monounsaturated fatty acid, *PUFA* polyunsaturated fatty acid, *ARA* arachidonic acid, *EPA* eicosapentaenoic acid, *DHA* docosahexaenoic acid

*Significant difference between visit and the previous one (*p* < 0.05)

#### SFA

The content of SFAs increased with increasing of carbon numbers in the FA, from butyric acid (4:0), which was at the limit of quantification (about 2 mg/100 mL milk) to palmitic acid (16:0) which was the most abundant SFA, at about 22–25% of the total FAs. The content of longer-chain SFAs (from 18:0 to 24:0) were lower than that of palmitic acid. Across lactation, the stearic acid (18:0) content stayed constant at ~ 6%, while caprylic (10:0), lauric (12:0), and myristic (14:0) acid compositions significantly increased between 0–3 days and 17 days. Palmitic, arachidic (20:0), and lignoceric (24:0) acid contents significantly decreased between 0–3 days and 17 days and remained stable until 120 days of lactation.

Therefore, a significant difference in the total SFA content was observed between 0 and 17 days and between 17 and 30 days, the total SFA content being higher in transitional milk than in colostrum and mature milk.

#### MUFA

Oleic acid (18:1 n-9) was the most abundant of all monounsaturated fatty acids (MUFAs) (34–36% of total FA) and of all FAs. Except for 16:1, the content of all MUFAs significantly decreased between 0–3 and 17 days. Eicosanoic (20:1 n-9), docosenoic (22:1 n-9), and nervonic acid (24:1 n-9) contents decreased over the lactation period (Table 2). A significant decrease in the total MUFA content was observed between 0 and 17 days, followed by a significant increase between 17 and 30 days.

#### PUFA

LA was the most abundant n-6 PUFA (12–14% of total FA) and ALA the most abundant n-3 PUFA (0.8–0.9%). Their content significantly increased between 0 and 17 days (from 12.2 to 13.2% for LA; from 0.81 to 0.85% for ALA) and then remained stable. The average ARA content in mature milk was 0.46% and average DHA was 0.36% (Supplementary Table 3). On the contrary of the 18 carbon precursors (LA and ALA), the two LCPUFAs ARA and DHA significantly decreased during lactation (from 0.91 to 0.44% and 0.68 to 0.34%, respectively, from day 0 to day 120), while EPA was stable at 0.12% of total FA. The ratio ARA: DHA ranged between 1.52 and 1.68 and was generally stable over lactation.

For most FA, similar trends were observed with concentration of FA (supplementary Table 1), despite the increase of total FA concentration from colostrum (1.92 g/100 mL) to mature milk (about 3 g/100 mL). One exception was the concentration of ARA and DHA, which were maximum in transitional milk, higher than in colostrum and mature milk.

### Plasma, erythrocytes, and adipose tissue fatty acids

The FA composition of maternal plasma and erythrocyte lipids (from venous blood) at delivery and of infant plasma and erythrocyte lipids (from cord blood) at birth is listed in Table 3 together with that of maternal adipose tissue at delivery.

**Table 3:** Plasma and erythrocytes (mother and infant) and adipose tissue (mothers) fatty acid composition (g/100 g of total FAs) (mean ± standard deviation)

	Plasma		Erythrocytes		Adipose tissue
Fatty acids (g/100 g of total FA)	Maternal *N* = 264	Infant (Cord Blood) *N* = 87	Maternal *N* = 264	Infant (Cord Blood) *N* = 87	Maternal *N* = 38
SFAs					
10:0	0.04 ± 0.04	0.05 ± 0.05	ND	ND	0.03 ± 0.01
12:0	0.11 ± 0.11	0.08 ± 0.04	ND -	ND	0.49 ± 0.16
13:0	-	-	0.11 ± 0.09	0.14 ± 0.17	0.02 ± 0.00
14:0	1.13 ± 0.45	0.80 ± 0.21	0.51 ± 0.19	0.43 ± 0.07	2.38 ± 0.51
14:0 iso	ND	ND	ND	ND	0.02 ± 0.01
15:0	0.22 ± 0.07	0.17 ± 0.05	ND	ND	0.23 ± 0.08
15:0 anteiso	ND	ND	ND	ND	0.05 ± 0.02
15:0 iso	0.02 ± 0.02	0.02 ± 0.12	ND	ND	0.03 ± 0.01
16:0	25.74 ± 1.98	26.32 ± 2.98	26.54 ± 5.59	30.44 ± 3.52	22.39 ± 1.78
16:0 iso	0.05 ± 0.02	0.07 ± 0.05	ND	ND	0.06 ± 0.02
17:0	0.23 ± 0.04	0.26 ± 0.07	0.33 ± 0.09	0.36 ± 0.20	0.22 ± 0.03
17:0 anteiso	0.13 ± 0.05	0.23 ± 0.06	ND	ND	0.14 ± 0.05
17:0 iso	ND	ND	ND	ND	0.05 ± 0.02
18:0	5.24 ± 0.85	9.37 ± 1.67	15.61 ± 3.33	21.46 ± 2.58	3.95 ± 0.66
20:0	0.22 ± 0.06	0.53 ± 0.12	0.46 ± 0.12	0.81 ± 0.12	0.13 ± 0.03
22:0	0.56 ± 0.11	1.01 ± 0.21	1.97 ± 0.42	2.09 ± 0.24	0.04 ± 0.01
23:0	0.28 ± 0.06	0.31 ± 0.07	ND	ND	ND
24:0	ND	ND	5.76 ± 1.44	7.60 ± 1.15	0.04 ± 0.01
26:0	ND	ND	0.37 ± 0.09	0.72 ± 0.14	ND
Total SFAs	34.82 ± 2.56	40.99 ± 4.61	61.96 ± 58.12	60.68 ± 20.91	30.31 ± 3.31
MUFAs					
14:1 n-5	0.06 ± 0.04	0.03 ± 0.03	ND	ND	0.20 ± 0.07
16:1 n-7	2.51 ± 0.79	3.43 ± 0.70	0.72 ± 0.30	0.65 ± 0.23	3.67 ± 0.82
16:1 n-9	1.05 ± 0.24	1.34 ± 0.39	ND	ND	0.53 ± 0.07
18:1 n-7	1.60 ± 0.25	2.77 ± 0.43	ND	ND	1.53 ± 0.15
18:1 n-9	21.48 ± 2.70	17.31 ± 2.38	13.30 ± 1.76	10.12 ± 1.64	40.74 ± 2.07
* trans*-18:1	0.31 ± 0.10	0.31 ± 0.16	0.36 ± 0.11	0.28 ± 0.10	0.48 ± 0.14
18:1 n-7	ND	ND	1.05 ± 0.18	1.77 ± 0.26	ND
20:1 n-9	0.23 ± 0.06	0.13 ± 0.08	0.28 ± 0.07	0.24 ± 0.07	0.60 ± 0.09
24:1 n-9	1.03 ± 0.23	1.48 ± 0.29	5.85 ± 0.81	5.05 ± 0.54	
Total MUFAs	27.94 ± 3.10	26.41 ± 0.97	25.05 ± 19.03	17.39 ± 6.97	46.74 ± 4.90
PUFAs					
18:2 n-6	24.23 ± 3.57	10.89 ± 3.72	7.52 ± 2.49	2.58 ± 1.27	19.60 ± 3.73
18:2 c/t	0.57 ± 0.20	0.43 ± 0.14	ND	ND	0.21 ± 0.05
20:2 n-6	0.25 ± 0.05	0.29 ± 0.09	ND	ND	0.30 ± 0.07
18:3 n-3	0.46 ± 0.30	0.10 ± 0.11	0.10 ± 0.08	0.03 ± 0.02	0.45 ± 0.12
18:3 n-6	0.23 ± 0.12	0.30 ± 0.09	0.07 ± 0.04	0.09 ± 0.04	0.08 ± 0.02
20:3 n-6	1.54 ± 0.36	2.76 ± 0.83	1.12 ± 0.56	1.30 ± 0.65	0.26 ± 0.09
20:4 n-6 (ARA)	5.40 ± 1.35	11.16 ± 3.38	6.88 ± 4.15	5.55 ± 3.41	0.39 ± 0.11
22:4 n-6	See below	See below	1.48 ± 1.05	1.13 ± 0.84	0.14 ± 0.06
22:4 + 24:0	0.61 ± 0.13	1.38 ± 0.28	Not relevant	Not relevant	Not relevant
20:5 n-3	0.32 ± 0.25	0.25 ± 0.21	0.21 ± 0.20	0.06 ± 0.05	0.04 ± 0.02
22:5 n-3	0.28 ± 0.11	0.28 ± 0.18	0.89 ± 0.69	0.25 ± 0.28	0.09 ± 0.04
22:5 n-6	0.31 ± 0.15	0.63 ± 0.32	0.41 ± 0.39	0.42 ± 0.36	0.04 ± 0.02
20:3 n-3	ND	ND	ND	ND	0.02 ± 0.01
22:6 n-3 (DHA)	2.36 ± 0.68	4.05 ± 1.63	3.24 ± 2.46	1.77 ± 1.61	0.13 ± 0.05
Total PUFAs	35.40 ± 4.15	30.71 ± 6.13	13.22 ± 10.17	13.73 ± 11.57	21.80 ± 4.17
Ratios					
ARA/DHA	2.63 ± 1.09	3.17 ± 1.45	2.82 ± 1.31	3.85 ± 1.20	3.31 ± 1.38
Total FAs (mg/100 mL)	462.3 ± 106.3	126.1 ± 73.6	148.2 ± 40.6	142. ± 45.1	not available

*SFA* saturated fatty acid, *MUFA* monounsaturated fatty acid, *PUFA* polyunsaturated fatty acid, *ND* not detected

18:1_b, not identified isomer of 18:1

#### Plasma

In plasma, as found in milk, palmitic acid was the most abundant SFA (26%, in mothers and infants) and oleic acid was the most abundant MUFA (21% in mothers, 17% in infants). LA was the major n-6 PUFA (24% in mothers, 11% in infants), but ARA was abundant as well (5.4% in mothers, 11.2% in infants). The most abundant n-3 PUFA was DHA (2% in mothers, 4% in infants), more abundant than in milk (0.68% at 0–3 days). The ratio ARA to DHA was 2.6 (mothers) and 3.2 (infants), higher than that observed in milk (~ 1.5).

#### Erythrocytes

As found in milk and plasma, palmitic acid was the most abundant SFA in erythrocytes (26% in mothers, 30% in infants) and oleic acid was the most abundant MUFA (13% in others, 10% in infants). Erythrocytes showed high amounts of stearic acid (15% in mothers, 21% in infants) and notable amounts of lignoceric, 24:0 (6% in mothers and 8% in infants) and nervonic acid, 24:1 n-6 (6% in mothers and 5% in infants). LA and ARA were the major n-6 PUFAs in erythrocytes: 7.5% (LA) and 6.9% (ARA) in mothers, and 2.6% (LA) and 5.6% (ARA) in infants. The most abundant n-3 PUFA was DHA (3.2% in mothers, 1.8% in infants). The ratio ARA to DHA was 2.8 (mothers) and 3.8 (infants), close to that observed in plasma but higher than what observed in milk (~ 1.5). The total SFA composition was about 61% in both mother and infant erythrocytes. Total MUFA composition was higher in mother (25%) erythrocytes than in infant (17%) one and PUFA composition comparable (about 13%) (Table 3).

#### Adipose tissue

Only few samples of adipose tissues from specific sites were obtained, i.e., France (*n* = 1), Portugal (*n* = 9), and Romania (*n* = 26). In adipose tissue the most abundant FA was oleic acid (40.7%) followed by palmitic and LA (22.4 and 19.6%, respectively). EPA, DHA, and ARA fractions were very low 0.04, 0.13%, and 0.39%, respectively (Table 3). The ARA to DHA ratio was 3.3, higher than what observed in milk and in maternal plasma and erythrocytes.

#### Maternal status vs infant status at birth

LA content was higher in maternal than in infant blood (24% vs. 11% in plasma; 7.5% vs. 2.6% in erythrocytes). Similarly, ALA content, although very low, was higher in maternal than in infant plasma (0.46 vs. 0.10%) and erythrocytes (0.10 vs. 0.03%). On the contrary, DHA and ARA contents were higher in infant plasma than in maternal plasma, while compositions in erythrocytes of mothers and infants were similar. The ratio ARA to DHA was higher in infant blood than in maternal blood. The content of other FAs were similar between maternal and infant blood (Table 3).

### Correlation between fatty acids levels in maternal plasma, erythrocytes, adipose tissue, and in human milk

Results of Spearman correlation (rho and *p* values) between milk FAs [colostrum, (0–3 days) transitional (17 ± 3 days), and mature (> 30 days)] and maternal plasma, erythrocytes, and adipose tissue FA are shown in Table 4. Correlations were calculated for the most abundant milk FAs (12–18 carbon SFAs, oleic acid) and the most physiologically important PUFAs (LA, ALA, ARA, EPA, and DHA). The correlation between plasma, erythrocytes, and adipose tissue LA and milk ARA was investigated; the correlations between plasma and erythrocytes ALA and milk EPA and DHA were investigated. When absolute values of rho ≥ 0.4 and *p* < 0.05, the correlation was considered significant. It should be noted that the number of observations used to calculate the correlation varies, *e.g.,* while DHA was measured in 266 colostrum samples, there were only 44 observations for EPA, because its small concentration made it undetectable in many samples. Only FA composition of milk measured in the same centers providing plasma and/or erythrocytes and/or adipose tissues were considered.

#### Correlation between fatty acid composition in human milk and in maternal plasma

**Fig. 3:**
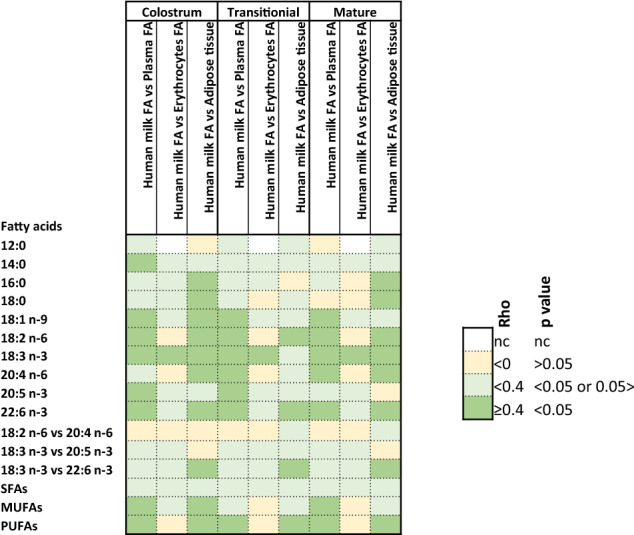
Mapping of correlation (rho/p) between fatty acid composition in human milk at different lactation stages (most abundant milk FAs and most physiologically relevant PUFAs were considered) and mother plasma, erythrocytes, and adipose tissue at delivery (Spearman correlation). In green are correlations considered to be relevant and significant because rho ≥ 0.4 and *p* < 0.05

The composition of individual SFA and of total SFAs in milk was not correlated to that of maternal plasma, erythrocytes, and adipose tissue, except for myristic in colostrum, which was positively correlated with myristic composition in maternal plasma (Fig. 3 and supplementary table 4). On the opposite, unsaturated FAs, i.e., oleic, LA, ALA, ARA, EPA (except in mature milk), DHA, total MUFA, and total PUFA composition in milk and plasma were positively correlated. LA composition in plasma did not correlate with ARA composition in milk. Similarly, ALA composition in plasma did not correlate with EPA or DHA in milk.

#### Correlation between fatty acids levels in human milk and in maternal erythrocytes

Contrary to the plasma FAs, erythrocyte FAs (myristic, palmitic, stearic, oleic, linoleic, EPA, DHA, total SFAs, MUFAs, and PUFAs) did not correlate with milk FAs, except for ALA (positive correlation). Neither ARA, EPA nor DHA composition in milk correlated with their precursor FA (LA for ARA; ALA for EPA and DHA) composition in erythrocytes.

#### Correlation between fatty acids levels in human milk and maternal adipose tissue

For most tested FAs, positive correlations were observed between colostrum and adipose tissue exception made for lauric, myristic, and total SFAs. Transitional milk FA compositions did not correlate with FA composition of adipose tissue, except for LA, DHA, and PUFAs while correlations were observed between mature milk and adipose tissue except for lauric, myristic, total SFAs, oleic acid, EPA, and total MUFAs. Finally, ALA composition in adipose tissue positively correlated with DHA composition in milk (colostrum, transitional, and mature).

### Correlation between fatty acids levels in maternal and cord blood at birth

**Fig. 4:**
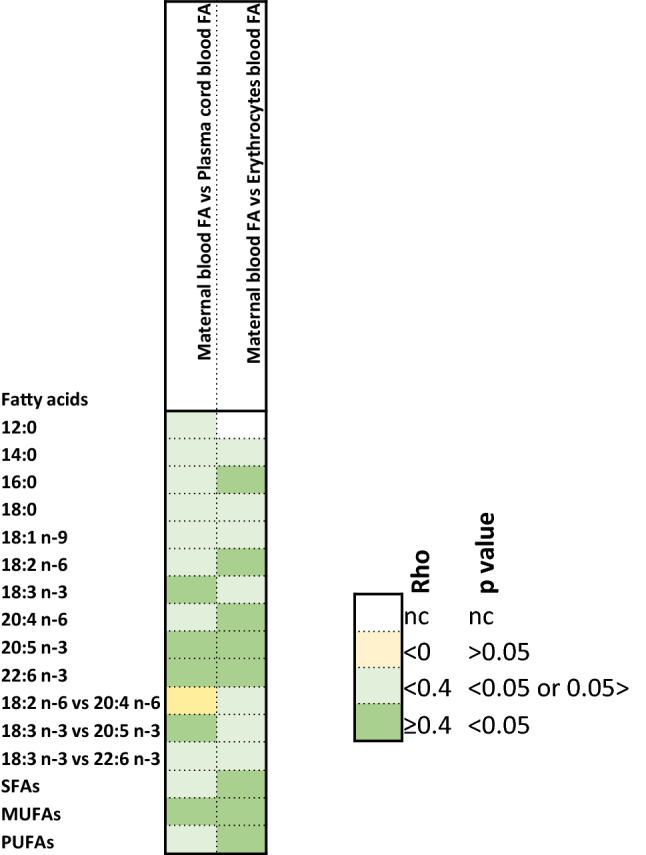
Mapping of correlation (rho/p) between fatty acid composition in maternal and cord blood plasma and erythrocytes at birth (Spearman correlation). In green are correlations considered to be relevant and significant because rho ≥ 0.4 and *p* < 0.05

Results of Spearman correlations between maternal and cord blood FAs (plasma and erythrocytes) at birth are shown in Fig. 4 and listed in supplementary table 5.

Between maternal and infant plasma, positive correlations were observed for ALA, LCPUFA n-3 (EPA or DHA) and for total MUFA compositions. In addition, EPA composition in infant plasma positively correlated with ALA composition in maternal plasma. More FAs correlated between mother and infant erythrocytes than for plasma, since palmitic acid, LA, ARA, EPA, DHA, total SFA, total MUFA, and total PUFA compositions were significantly correlated.

## Discussion

The present study was designed to assess the FA profile of full expressed HM collected in Norway, Sweden, Romania, France, Portugal, Spain, and Italy over first four months of lactation and to evaluate the correlation between the maternal FA status (plasma, erythrocyte, adipose tissue) and milk FA, and between the maternal blood FAs and infant blood FAs at birth and to evaluate factors influencing FA composition of HM. To our knowledge this is the first study combining the HM analysis with FA measurements in maternal blood (plasma and erythrocytes) and adipose tissue and cord blood (plasma and erythrocytes) in the same cohort. Our findings showed major difference in FA HM composition between colostrum (0–3 days after delivery) and transitional milk (17 ± 5 days after delivery) and minor differences between transitional milk and mature milk. Mature milk FA composition was generally constant over lactation. Maternal plasma and adipose tissue FA composition showed correlation with HM one, while maternal erythrocyte FA was not correlated with milk FAs. Positive correlations were observed for EPA and DHA composition between maternal and infant blood. The essential FAs LA and ALA compositions were higher in maternal than in infant blood, while ARA and DHA compositions were low in maternal blood supporting biomagnification of LCPUFAs in this population, i.e., the potential preferential transfer of DHA and ARA from the mother to the infant. Total fat in colostrum was 2.7 g/100 ml and about 4 g/100 ml in transitional and mature milk in agreement with previous studies [1–3].

The general pattern of the FA composition of HM observed in this study (compositions of short-, medium-, and odd-chain saturated, as well as of MUFAs and PUFAs) agreed with what was previously reported in different countries [14]. Oleic acid was the most abundant FA (35–38%) followed by palmitic acid (22–25%) (Table 2) in agreement with previous works [21, 22, 34–39]. LA composition (12–14%) was comparable to that reported in HM from European women [13, 38–41], and relatively lower than what reported in milk of Chinese and South American population [13, 35–37, 42], traditionally consuming vegetable oils rich in LA, such as soybean and sunflower oils [42, 43]. Same as LA, the composition of ALA (0.8–0.9%) was lower than in other countries like China where soybean oil, rich in ALA, is one of the main sources of fat [43]. The composition of the essential fatty acid ALA also increased slightly with progression of lactation from colostrum (0.8%) to mature milk (0.9%) which is in line with previous observations in Europe and Asia [21, 35–40, 44]. On the other hand, Guesnet [16] in a study performed in France showed constant ALA composition across lactation at level of 0.6%.

In this study, ARA composition in mature milk ranged between 0.44 and 0.50%, which is comparable to what found by Li et al. [42] (0.56% ± 0.31) for European countries and Brenna [45] for non-European countries, but lower than the range for ARA (0.7–1.1%) established by EFSA as “typical milk composition in Europe” on the basis of values measured in Greece and Finland [41]. These differences may be due to the difference in the included countries, Greece and Finland in EFSA report.

In this study, the DHA composition in human milk across the 7 countries ranged between 0.34 and 0.40%, in agreement with Li et al. [42] and EFSA [40] (0.34% ± 0.14, 0.2–0.5%, respectively) for Europe and with Brenna [45] for non-European countries. ARA and DHA compositions decreased from 0.9 to 0.4% and from 0.7 to 0.3%, respectively, between day 0 and day 120, in agreement with previous studies [12, 21, 22, 35, 38–40]. The ARA to DHA ratio (1.47–1.68) was lower than the value of 1.89 reported by Li et al. [42], which included the high ARA value of Switzerland. Recently EFSA [46] recommended only the addition of DHA to infant formula, which may lead to an ARA/DHA ratio lower than what is reported in this study and others [47] and recommended by Early Nutrition Academy [48]. The EPA composition was constant along lactation (0.1%) as previously reported by Chen and Genzel-Boroviczeny [35, 38].

ARA, EPA, and DHA can be formed from their respective precursors LA and ALA. The conversion of LA to ARA and ALA to EPA and DHA in man, when estimated on the basis of the appearance of products of stable isotope tracers in plasma, is < 1% [7]. LCPUFA composition of the tissues of breastfed infants depend largely on the composition found in their mothers’ milk, and therefore on the mother’s diet. In this study, milk DHA composition was lower than what was reported in coastal populations [13, 37, 49], which have easier access to fish, the main dietary sources of EPA and DHA.

We explored different variables (lactation stage, country, birth parity, birth gestational age, maternal BMI, gender) which are important in explaining the FA concentrations and compositions in milk, by mixed linear modeling and performed an analysis of variance on the model. The lactation stages and countries appeared to consistently be significant covariates. For a few FAs, the parity of birth was significant as well as the gestational age at birth. Since country was an important covariate, further subgroup correlations between the different sources of FAs were performed per country. However, due to the low number of observations for some countries, the correlations were not consistent among the different countries (results not shown). FA concentrations and composition of HM from the seven European countries are shown in supplementary tables.

In maternal plasma collected within 72 h from delivery, palmitic, oleic, and LA were the most abundant FAs in agreement with previous studies in Afro-American and German women [50, 51]. This is in line with high milk concentration of these three FAs, as plasma FAs can readily be transferred to milk. In our study, DHA composition in plasma (2.36% ± 0.68) was higher than what was found by Stark et al*.* [50] (1.86% ± 0.40) or Enke et al. [51] (1.21 ± 0.35) who reported fish consumption less than once a week in Afro-American or German women or by Oliveira et al. [52] (1.5 ± 0.6) who reported low consumption of n-3 PUFA food sources in Brazilian women. LCPUFA supplementation (e.g., by fish oil capsules) was not common in the mothers from the US or Germany [50, 51], as in the current study, where only about 5% of the mothers had DHA supplements during lactation (Supplementary Table 6).

In maternal erythrocytes collected 72 h after delivery, palmitic, stearic, and oleic were the most abundant FA, which agreed with previous studies [50, 51]. Erythrocyte DHA (3.24% ± 2.46) was higher in Afro-American women (1.41% ± 0.24) [49], similar to that of German women (3.40% ± 1.45) [51] and lower in mothers from Dominica (6.40% ± 0.59) [53], Africa (4–8%) [25], or Japan (7.41%) [54], which may be related to the respective intake of n-3 DHA food sources of these different populations. The FA profile of erythrocytes is a combined picture of interactions between dietary intake and stored FAs attributed to long half-life of erythrocytes (120 days) [55, 56]. Erythrocytes FA profile is generally considered to represent long-term (few weeks to few months) FA intakes, while plasma FA profile reflects short-term FA intake [19, 57].

In maternal adipose tissue, the LA concentration was higher in colostrum, whereas ARA, ALA, EPA, and DHA were lower, consistent with Martin et al*.* [58] and Luxwolda et al. [25] and suggesting selective uptake of ALA and LCPUFAs from body stores to milk.

Our study is the first to assess the correlation among FAs in milk and in maternal plasma, erythrocytes, and adipose tissue. Regarding unsaturated FAs, more positive correlations were found between mothers’ plasma or adipose tissue and HM than between maternal erythrocyte and HM, suggesting that short-term diet and lipid storage pools with slow turn-over, such as adipose tissue, influence unsaturated FA composition in HM more than circulating cells, such as erythrocytes [59].

Major differences between maternal and infant (cord blood) plasma were observed for DHA and ARA compositions which were almost double in infant’s plasma than in mothers one at birth. On the contrary, LA and ALA compositions were lower in infant’s plasma compared to maternal ones. Similar observations have been reported in different geographies [23, 51–53, 60–63]. LA and ALA obtained by the diet can be further metabolized to form ARA and EPA, respectively. Two successive elongations of EPA form 24:5n-3; then desaturation at position 6 yields to 24:6n-3, which is then β-oxidized to generates DHA [24, 64]. In the placenta, the activity of desaturases may be low as no desaturase activity was detected in human placenta microsomes [65], while other studies revealed that human placenta expressed ∆5- and ∆6-desaturase mRNA, although less than other tissues, like liver [66, 67]. Fetal LC-PUFA status is also determined to some extent by fetal fatty acid conversion [68]. Finally, ARA and DHA in cord blood plasma is derived from the mother by transfer through placenta [25, 68, 69]. Indeed, Haggarty et al. [70] found a selective preferential transport of DHA across the placenta with order of preference DHA > ARA > ALA > LA.

Differences in some PUFA compositions were observed between maternal and cord blood erythrocytes. As for plasma, LA and ALA compositions were higher in maternal than in cord blood erythrocytes, consistent with other studies [51, 52, 54, 63]. However, in our population, ARA and DHA compositions were higher in mothers than in cord blood erythrocytes, in disagreement with what observed in Polish, German or Japanese mothers with either lower or higher DHA status [51, 54, 63] than in the current European cohort. A study performed in African mothers and infants showed that infants with lower DHA status had higher erythrocyte DHA composition than their mothers [25], in agreement with the hypothesis that intra-uterine DHA biomagnification would be a sign of low DHA status.

Positive and significant correlations (rho ≥ 0.4 and *p* value < 0.05) were observed between maternal and cord plasma for ALA, EPA, and DHA compositions and between maternal and cord erythrocytes for ARA, EPA, and DHA, supporting the transfer of these FAs across the placenta [23, 54, 63, 69]. No correlation was observed among SFAs (12:0–18:0) compositions in milk and maternal plasma and erythrocytes in agreement with other studies [51, 54]. This may be explained by the presence in the cytosol of mammary epithelial cells of a medium-chain acylthioester hydrolase, thioesterase II, that terminates chain elongation at 8–14 carbons [67], resulting in the de novo synthesis of medium-chain FAs. On the other hand, long-chain saturated (≥ 16:0) and unsaturated FAs enter the mammary alveolar cell from the plasma and are derived from the diet or from lipid stores [5, 67].

Positive correlations were observed for all FAs between colostrum or mature milk and adipose tissue exception made for 12:0 and 14:0, supporting the de novo synthesis of these FAs in the mammary gland and that FAs ≥ 16 (saturated or unsaturated) are either mobilized form stores, such as adipose tissue or derived from the diet [4, 58]. Positive correlations were observed between milk and plasma for unsaturated FAs in agreement with previous findings [23]. If mobilized from mother stores, the lack of correlation in FAs between mother erythrocytes, adipose tissue, and mature milk may indicate the need of supplementation in order to rebuild PUFA and LCPUFA store in mother during lactation. However, these are observations based on associations and further investigation using labeled molecules is needed to demonstrate it.

The limitations of the current study include lack of availability of dietary data, the impossibility to make country wise comparisons due to limited sample size in few countries, availability of pregnancy weight gain due to enrollment time, and unavailability of longitudinal data on infant and maternal FA status. In addition, adipose tissue measurements were available only in a small sub-set of samples. The strength of this study is that milk was collected by complete breast emptying, ensuring more homogeneity in the milk quality, that FAs were measured in the same population in different maternal body compartments (plasma, erythrocytes and adipose tissue) and infants tissues (plasma, erythrocytes), allowing multiple comparisons and correlations between mothers–infants pairs. Finally, the enrollment of mothers from 7 European countries and the number of milk samplings across lactation allowed to provide a typical profile and composition of FAs in European human milk.

## Conclusions

Our findings showed that adipose tissue rather than erythrocytes may serve as reservoir of PUFAs and LCPUFAs for human milk. Plasma also supplies PUFAs and LC-PUFAs to the lactating mother. If both, adipose tissue and plasma PUFAs, are reflection of dietary intake, it is necessary to provide sufficient amount of LCPUFAs during pregnancy to ensure their provision to infant and mother. Finally, these data on mature milk FA composition measured on over 200 women in 7 European countries at 4 time points across lactation could be used to build a typical composition of mature milk in European women.

## Supplementary Information

Below is the link to the electronic supplementary material.

Supplementary file1 (DOCX 115 kb)
