# Supplementary material for: Human milk fatty acid composition and its association with maternal blood and adipose tissue fatty acid content in a cohort of women from Europe
Source: Eur J Nutr. 2022 Jan 24;61(4):2167–82. doi: 10.1007/s00394-021-02788-6 (PMC9106604; doi:10.1007/s00394-021-02788-6)
Supplement: Supplementary file 1 — Supplementary file1 (DOCX 115 kb) [file 394_2021_2788_MOESM1_ESM.docx]

**Supplementary Tables**

**Supplementary table 1. Human milk fatty acid concentration (mg/100 mL) in human milk at different lactation stages (Mean ±standard deviation) and number of observations.**

|  | Mean± SD | Mean± SD | Mean± SD | Mean± SD | Mean± SD | Mean± SD |
| --- | --- | --- | --- | --- | --- | --- |
| **Fatty acids (mg/100ml)** | **0-3 d** | **17±3 d** | **30±3 d** | **60±5 d** | **90±5 d** | **120±5 d** |
| **SFAs** |  |  |  |  |  |  |
| 6:0 | 2.75 ± 0.72 (2) | 2.44±0.34 (26) | 2.54±0.45 (53) | 2.72±0.61(88) | 2.95± 0.94 (101) | 3.09±1.04 (107) |
| 8:0 | 5.44±4.01 (22) | 6.66±2.84 (253) | 6.88±2.90 (225) | 6.66±3.03 (216) | 6.70±3.32 (209) | 6.82±3.34 (191) |
| 10:0 | 10.11±13.65 (232) | 47.78±20.64 (292) | 45.93±20.55 (225) | 43.54±21.00 (241) | 43.05±24.23 (223) | 43.10±24.34 (223) |
| 12:0 | 52.11±53.56 (232) | 174.61± 85.46* (292) | 160.15±80.01 (225) | 153.19±89.28 (241) | 160.02±99.52 (232) | 165.10±106.10 (223) |
| 14:0 | 105.34±69.25 (232) | 186.50±90.66 (292) | 173.73± 90.22*(225) | 169.35±92.87 (241) | 178.59±106.17 (223) | 186.77±114.93 (223) |
| 16:0 | 481.39±250.77 (269) | 667.30± 274.01* (292) | 686.56±309.98 (261) | 672.46±309.06 (241) | 660.01±342.67 (232) | 663.46±354.69 (223) |
| 18:0 | 116.64±64.92 (232) | 185.98± 89.59* (292) | 195.45±94.23 (225) | 205.13±103.84 (241) | 195.40±108.37 (232) | 200.92±114.78 (223) |
| 20:0 | 4.52±2.09 (246) | 5.65± 2.55* (277) | 5.66±2.36 (243) | 5.85±2.71 (220) | 5.60±2.65 (207) | 5.79±2.80 (194) |
| 24:0 | 7.07±3.54 (251) | 3.60±1.35 (203) | 3.04± 0.94* (148) | 3.01±0.96 (102) | 2.91±1.02 (81) | 3.09±1.21 (87) |
| **MUFAs** |  |  |  |  |  |  |
| 16:1 n-7 | 39.70±24.88 (232) | 70.83±36.02 (292) | 75.99±39.47 (225) | 72.12±39.84 (241) | 67.79±41.69 (232) | 67.40±41.50 (223) |
| 18:1 n-7 | 58.58±32.68 (232) | 74.97± 34.86* (292) | 75.58±35.91 (225) | 74.87±40.10 (241) | 70.99±41.23 (232) | 72.27±41.09 (223) |
| 18:1 n-9 | 683.63±348.78 (269) | 1032.36± 452.16* (292) | 1098.70± 495.18* (261) | 1136.51±567.22 (241) | 1084.34±585.35 (232) | 1086.01±590.02 (223) |
| *trans*-18:1 | 11.21±6.95 (261) | 18.33±11.44 (292) | 18.82±12.12 (261) | 20.04±13.77 (240) | 18.96±12.30 (231) | 19.47±13.95 (222) |
| 20:1 n-9 | 16.56±8.83 (268) | 14.40± 7.44* (292) | 14.03±7.78 (261) | 13.06±8.08 (240) | 12.20±8.29 (228) | 12.22±7.65 (217) |
| 22:1 n-9 | 4.45±2.04 (236) | 3.51± 1.33* (197) | 3.37± 1.52* (157) | 3.41±1.60 (108) | 3.26±1.40 (91) | 3.23±1.29 (93) |
| 24:1 n-9 | 7.07±3.54 (266) | 3.60±1.35 (215) | 3.04± 0.94* (147) | 3.01±0.96 (89) | 2.91±1.02 (74) | 3.04±1.04 (66) |
| **PUFAs** |  |  |  |  |  |  |
| 18:2 n-6 | 234.49±145.62 (269) | 390.84±204.78 (292) | 416.12±252.85 (261) | 433.99±286.18 (241) | 422.37±293.19 (232) | 418.87±273.02 (223) |
| 18:3 n-3 | 15.70±11.30 (264) | 26.42±21.31 (269) | 27.63±22.17 (261) | 31.13±31.06 (241) | 27.64±25.62 (232) | 29.27±27.16 (221) |
| 18:3 n-6 | 3.430±1.43 (20) | 3.978±2.23 (197) | 4.50±2.42 (195) | 4.36±1.92 (180) | 4.29±2.15 (163) | 4.36±2.13 (144) |
| 20:2 n-6 | 15.09±8.84 (266) | 11.42± 5.78* (292) | 10.33± 5.59* (260) | 8.87± 5.07* (240) | 8.40±5.08 (221) | 8.26±4.56 (212) |
| 20:3 n-6 | 13.22±8.05 (267) | 14.29± 7.46* (292) | 13.44±7.07 (260) | 11.09± 5.81* (241) | 10.05± 5.79* (225) | 9.65±5.61 (216) |
| 20:4 n-6 | 16.42±9.03 (269) | 16.91± 7.40*(292) | 15.12± 7.42* (261) | 13.90± 7.09* (241) | 13.13±7.59 (231) | 13.39±8.33 (219) |
| 20:5 n-3 | 3.15±1.47 (44) | 4.04±2.24 (131) | 4.20±3.16 (121) | 4.28±2.73 (103) | 3.95±2.79 (102) | 4.49±4.67 (87) |
| 22:6 n-3 | 12.83±8.47 (266) | 14.17± 9.83* (291) | 12.53± 9.60* (257) | 10.94±7.72 (233) | 10.35±8.07 (222) | 10.55±13.24 (212) |
| ARA/DHA | 1.52±0.76 (269) | 1.47±0.75 (292) | 1.52±0.75 (261) | 1.62±0.77 (241) | 1.59±0.81 (232) | 1.68±0.89 (223) |
| EPA+DHA | 15.98±8.59 | 18.22±10.08 | 16.72±10.10 | 15.22±8.19 | 14.30±8.54 | 15.04±14.04 |
| Total SFAs (g/100ml) | 0.77±0.43 | 1.28±0.51 | 1.28±0.56 | 1.26±0.58 | 1.25±0.65 | 1.27±0.67 |
| Total MUFAs (g/100ml) | 0.82±0.42 | 1.22± 0.53* | 1.29±0.57 | 1.32±0.65 | 1.26±0.68 | 1.26±0.68 |
| Total PUFAs (g/100ml) | 0.31±0.18 | 0.48± 0.24* | 0.50±0.28 | 0.52±0.32 | 0.50±0.33 | 0.49±0.31 |
|  |  |  |  |  |  |  |

** significant difference between visit and the previous one. (p<0.05)*

**Supplementary table 2a. Fatty acid composition (g/100g of total FA) of colostrum (0±3 days after delivery) obtained from women in 7 European countries (mean ± standard deviation (number of observations).**

| Fatty acids (g/100g total FA) | **Spain** | **France** | | **Italy** | | **Norway** | | **Portugal** | | **Romania** | | **Sweden** | |
| --- | --- | --- | --- | --- | --- | --- | --- | --- | --- | --- | --- | --- | --- |
| COLOSTRUM | Mean±std (N) | Mean±std (N) | | Mean±std (N) | | Mean±std (N) | | Mean±std (N) | | Mean±std (N) | | Mean±std (N) | |
| **SFAs** |  |  | |  | |  | |  | |  | |  | |
| 6:0 | 0.06 (1) | 0.06 (1) | | - | | - | | - | | - | | - | |
| 8:0 | 0.14±0.06 (2) | 0.18±0.12 (6) | | - | | 0.05 (1) | | 0.17±0.06 (4) | | 0.21±0.08 (6) | | 0.10±0.02 (3) | |
| 10:0 | 0.49±0.33 (7) | 0.54±0.30 (79) | | 0.40±0.18 (8) | | 0.32±0.13 (7) | | 0.32±0.27 (70) | | 0.58±0.58 (29) | | 0.39±0.18 (32) | |
| 12:0 | 2.52±1.27 (10) | 2.90±1.24 (80) | | 2.35±0.83 (8) | | 2.41±0.71 (9) | | 2.04±1.05 (87) | | 2.72-2.04 (38) | | 2.57±0.77 (37) | |
| 14:0 | 4.64±1.83 (10) | 6.18±1.44 (80) | | 4.92±0.80 (8) | | 5.70±1.16 (9) | | 4.71±1.10 (87) | | 4.68±1.61 (38) | | 6.29±1.27 (37) | |
| 16:0 | 21.18±2.70(10) | 25.29±2.07 (80) | | 24.92±1.47(8) | | 25.09±1.57(9) | | 26.05±1.88(87) | | 24.99±2.23 (38) | | 25.76±2.54 (37) | |
| 18:0 | 5.56±1.06 (10) | 6.49±1.18 (80) | | 5.30±1.12 (8) | | 7.85±1.31 (9) | | 5.66±0.76 (87) | | 6.04±1.02 (38) | | 6.99±1.23 (37) | |
| 20:0 | 0.22±0.07 (9) | 0.25±0.10 (78) | | 0.18±0.06 (6) | | 0.36±0.13 (9) | | 0.22±0.07 (76) | | 0.22±0.06 (34) | | 0.27±0.06 (34) | |
| 24:0 | 0.23±0.12 (10) | 0.24±0.13 (78) | | 0.17±0.06 (6) | | 0.37±0.22 (9) | | 0.25±0.12 (78) | | 0.25±0.13 (36) | | 0.25±0.08 (34) | |
| **MUFAs** |  |  | |  | |  | |  | |  | |  | |
| 16:1 n-7 | 1.46±0.43 (10) | 2.20±0.61 (80) | | 2.36±0.55 (8) | | 1.80±0.67 (9) | | 2.06±0.61 (87) | | 1.89±0.44 (38) | | 1.93±0.55 (37) | |
| 18:1 n-7 | 2.85±0.45 (10) | 2.76±0.38 (80) | | 3.76±0.43 (8) | | 3.18±0.41 (9) | | 3.36±0.49 (87) | | 2.67±0.48 (38) | | 3.45±0.63 (37) | |
| 18:1 n-9 | 40.92±4.42(10) | 37.04±2.65 (80) | | 37.99±1.87(8) | | 36.01±1.78 (9) | | 35.12±2.72 (87) | | 33.99±2.98 (38) | | 36.73±2.84 (37) | |
| *trans*-18:1 | 8.36±6.44 (10) | 11.97±6.33 (80) | | 10.65±4.64 (7) | | 13.23±7.70 (8) | | 9.07±5.26 (83) | | 11.71±10.03 (38) | | 14.46±6.83 (35) | |
| 20:1 n-9 | 0.87±0.23 (10) | 0.91±0.16 (80) | | 0.86±0.21 (8) | | 1.11±0.24 (9) | | 0.83±0.16 (87) | | 0.77±0.20 (38) | | 1.04±0.23 (37) | |
| 22:1 n-9 | 0.23±0.08 (10) | 0.22±0.05 (74) | | 0.21±0.05 (7) | | 0.28±0.08 (8) | | 0.22±0.05 (72) | | 0.18±0.05 (30) | | 0.26±0.07 (35) | |
| 24:1 n-9 | 0.41±0.20 (10) | 0.38±0.15 (80) | | 0.37±0.10 (7) | | 0.56±0.26 (9) | | 0.42±0.16 (86) | | 0.39±0.22 (38) | | 0.44±0.14 (36) | |
| **PUFAs** |  |  | |  | |  | |  | |  | |  | |
| 18:2 n-6 | 14.71±2.55(10) | 9.96±1.62 (80) | 12.09±1.83(8) | | 9.75±1.26 (9) | | 14.03±1.98 (87) | | 16.29±2.41 (38) | | 8.91±1.96 (37) | |  |
| 20:2 n-6 | 0.86±0.30 (10) | 0.63±0.14 (79) | | 0.77±0.17 (7) | | 0.73±0.15 (9) | | 0.94±0.20 (87) | | 1.06±0.30 (38) | | 0.63±0.17 (36) | |
| 18:3 n-3 | 0.45±0.17 (10) | 1.03±0.33 (80) | | 0.65-0.20 (8) | | 1.10±0.26 (9) | | 0.59±0.17 (85) | | 0.43±0.12 (36) | | 1.25±0.35 (36) | |
| 18:3 n-6 | 0.14±0.00 (2) | 0.10±0.01 (3) | | 0.11±0.03 (3) | | - | | 0.09±0.07 (6) | | 0.11±0.06 (6) | | - | |
| 20:3 n-6 | 0.61±0.18 (10) | 0.61±0.15 (80) | | 0.74±0.24 (7) | | 0.66±0.20 (9) | | 0.85±0.23 (87) | | 0.77±0.23 (38) | | 0.57-0.15 (36) | |
| 20:4 n-6 | 0.71±0.18 (10) | 0.85±0.20 (80) | | 0.79±0.17 (8) | | 0.84±0.24 (9) | | 1.03±0.25 (87) | | 1.01±0.33 (38) | | 0.72±0.21 (37) | |
| 20:5 n-3 | 0.09 (1) | 0.12±0.04 (18) | | - | | 0.19±0.10 (4) | | 0.10±0.04 (12) | | 0.16 (1) | | 0.10±0.04 (8) | |
| 22:6 n-3 | 0.49±0.17 (10) | 0.71±0.23 (80) | | 0.62±0.18 (7) | | 1.07±0.42 (9) | | 0.74±0.25 (87) | | 0.41±0.17 (36) | | 0.73±0.28 (37) | |
| ARA/DHA | 1.53±0.41 (10) | 1.26±0.34 (80) | | 1.55±0.60 (8) | | 0.92±0.44 (9) | | 1.49±0.44 (87) | | 2.73±1.12 (38) | | 1.06±0.32 (37) | |
| Total SFAs | 34.95 ± 4.48 (10) | 41.92 ± 4.09 (80) | | 38.3 ± 1.52 (8) | | 42.11 ± 2.81 (9) | | 39.27 ± 2.94 (87) | | 39.44 ± 3.71 (38) | | 42.56 ± 4.07 (37) | |
| Total MUFAs | 47.12 ± 4.64 (10) | 44.15 ± 3.04 (80) | | 46.05 ± 2.32 (8) | | 43.58 ± 1.84 (9) | | 42.47 ± 2.91 (87) | | 40.52 ± 3.63 (38) | | 44.53 ± 3.11 (37) | |
| Total PUFAs | 17.93 ± 2.63 (10) | 13.91 ± 1.94 (80) | | 15.65 ± 1.94 (8) | | 14.31 ± 1.74 (9) | | 18.26 ± 2.25 (87) | | 20.04 ± 2.48 (38) | | 12.91 ± 2.14 (37) | |

**Supplementary table 2b. Fatty acid composition (g/100g of total FA) of transitional milk (17±5 days after delivery) obtained from women in 7 European countries (mean ± standard deviation (number of observations).**

|  | **ESP** | **FRA** | **ITA** | **NOR** | **PRT** | **ROU** | **SWE** |
| --- | --- | --- | --- | --- | --- | --- | --- |
| Fatty acids (g/100g) | **Transitional** | **Transitional** | **Transitional** | **Transitional** | **Transitional** | **Transitional** | **Transitional** |
| TRANSITIONAL | Mean±std (N) | Mean±std (N) | Mean±std (N) | Mean±std (N) | Mean±std (N) | Mean±std (N) | Mean±std (N) |
| **SFAs** |  |  |  |  |  |  |  |
| 6:0 | - | 0.06±0.01 (10) | - | 0.07(1) | 0.05±0.01 (4) | 0.06±0.02 (8) | 0.05±0.03 (3) |
| 8:0 | 0.22±0.058 (8) | 0.26±0.07 (50) | 0.22±0.05 (12) | 0.21±0.04 (10) | 0.21±0.05 (94) | 0.24±0.08 (39) | 0.22±0.06 (40) |
| 10:0 | 1.51±0.49 (9) | 1.72±0.42 (84) | 1.65±0.41 (13) | 1.50±0.27 (10) | 1.61±0.35 (95) | 1.67±0.55 (40) | 1.56±0.39 (41) |
| 12:0 | 5.83±1.59 (9) | 5.72±1.89 (84) | 5.99±1.79 (13) | 5.26±1.42 (10) | 6.19±2.00 (95) | 6.54±2.52 (40) | 5.61±1.91 (41) |
| 14:0 | 5.45±1.38 (9) | 6.71±1.72 (84) | 6.43±1.67 (13) | 5.53±1.16 (10) | 6.02±2.09 (95) | 6.48±2.33 (40) | 6.64±2.01 (41) |
| 16:0 | 19.11±1.96 (9) | 23.34±2.29 (84) | 22.75±2.09 (13) | 21.13±1.98 (10) | 22.32±2.53 (95) | 22.67±2.76 (40) | 22.10±3.29 (41) |
| 18:0 | 5.76±0.59 (9) | 7.29±1.41 (84) | 5.96±1.74 (13) | 6.76±1.41 (10) | 5.27±0.87 (95) | 5.73±0.89 (40) | 6.80±1.39 (41) |
| 20:0 | 0.19±0.03 (9) | 0.22±0.04 (82) | 0.17±0.04 (11) | 0.22±0.10 (10) | 0.16±0.06 (88) | 0.17±0.04 (37) | 0.19±0.04 (40) |
| 24:0 | 0.14±0.10 (7) | 0.11±0.02 (66) | 0.09±0.02 (7) | 0.10±0.05 (10) | 0.10±0.02 (59) | 0.11±0.05 (27) | 0.08±0.02 (27) |
| **MUFAs** |  |  |  |  |  |  |  |
| 16:1 n-7 | 1.42±0.36 (9) | 2.44±0.64 (84) | 2.32±0.60 (13) | 2.63±0.79 (10) | 2.48±0.71 (95) | 2.17±0.57 (40) | 2.43±0.67 (41) |
| 18:1 n-7 | 2.43±0.52 (9) | 2.17±0.33 (84) | 2.80±0.45 (13) | 2.65±0.43 (10) | 2.70±0.39 (95) | 2.28±0.51 (40) | 2.88±0.57 (41) |
| 18:1 n-9 | 38.17±4.79 (9) | 35.63±3.18 (84) | 36.12±3.67 (13) | 36.96±2.50 (10) | 33.43±4.08 (95) | 30.34±2.85 (40) | 36.46±4.55 (41) |
| *trans*-18:1 | 11.70±5.69 (9) | 21.97±10.00 (84) | 15.24±10.11 (13) | 20.37±9.50 (10) | 12.83±7.58 (95) | 19.75±16.42 (40) | 24.17±11.76 (41) |
| 20:1 n-9 | 0.52±0.15 (9) | 0.51±0.13 (84) | 0.47±0.17 (13) | 0.59±0.13 (10) | 0.42±0.10 (95) | 0.39±0.08 (40) | 0.57±0.13 (41) |
| 22:1 n-9 | 0.12±0.07 (7) | 0.11±0.03 (64) | 0.10±0.04 (6) | 0.11±0.0 (9) | 0.09±0.03 (54) | 0.09±0.03 (23) | 0.11±0.03 (34) |
| 24:1 n-9 | 0.16±0.15 (7) | 0.11±0.03 (65) | 0.11±0.03 (8) | 0.10±0.02 (9) | 0.11±0.04 (63) | 0.12±0.06 (30) | 0.11±0.03 (33) |
| **PUFAs** |  |  |  |  |  |  |  |
| 18:2 n-6 | 15.50±2.62 (9) | 10.20±2.30 (84) | 11.76±2.73 (13) | 12.08±2.54 (10) | 15.42±3.66 (95) | 17.52±3.69 (40) | 10.25±2.93 (41) |
| 20:2 n-6 | 0.52±0.23 (9) | 0.31±0.06 (84) | 0.33±0.06 (13) | 0.34±0.07 (10) | 0.44±0.10 (95) | 0.52±0.14 (40) | 0.30±0.09 (41) |
| 18:3 n-3 | 0.71±0.57 (9) | 0.93±0.34 (84) | 0.59±0.19 (13) | 1.37±0.48 (10) | 0.65±0.28 (95) | 0.50±0.20 (40) | 1.50±0.58 (41) |
| 18:3 n-6 | 0.16±0.03 (7) | 0.10±0.04 (47) | 0.12±0.05 (7) | 0.10±0.04 (8) | 014±0.06 (69) | 0.14±0.05 (40) | 0.09±0.03 (29) |
| 20:3 n-6 | 0.63±0.22 (9) | 0.41±0.10 (84) | 0.44±0.14 (13) | 0.40±0.10 (10) | 0.57±0.16 (95) | 0.56±0.16 (40) | 0.37±0.08 (41) |
| 20:4 n-6 | 0.54±0.11 (9) | 0.53±0.09 (84) | 0.51±0.06 (13) | 0.50±0.07 (10) | 0.65±0.12 (95) | 0.70±0.16 (40) | 0.44±0.09 (41) |
| 20:5 n-3 | 0.16 (1) | 0.11±0.08 (45) | 0.17±0.08 (4) | 0.18±0.08 (9) | 0.11±0.07 (30) | 0.09±0.03 (9) | 0.12±0.05 (33) |
| 22:6 n-3 | 0.45±0.22 (9) | 0.49±0.30 (84) | 0.42±0.21 (13) | 0.64±0.27 (10) | 0.51±0.23 (95) | 0.32±0.15 (39) | 0.47±0.17 (41) |
| ARA/DHA | 1.38±0.45 (9) | 1.26±0.42 (84) | 1.52±0.80 (13) | 0.87±0.29 (10) | 1.45±0.55 (95) | 2.56±1.06 (40) | 1.04±0.32 (41) |
| Total SFAs | 38.21±4.07 (9) | 45.27±4.75 (84) | 43.29±4.68 (13) | 40.76±3.09 (10) | 41.90±5.09 (95) | 43.64±5.34 (40) | 43.22±6.31 (41) |
| Total MUFAs | 43.27±4.42 (9) | 41.68±3.40 (84) | 42.48±4.17 (13) | 43.65±2.88 (10) | 39.68±4.32 (95) | 36.07±3.52 (40) | 43.26±4.78 (41) |
| Total PUFAs | 18.53±2.87 (9) | 13.05±2.65 (84) | 14.22±2.70 (13) | 15.59±2.61 (10) | 18.43±3.76 (95) | 20.29±3.80 (40) | 13.51±3.60 (41) |

**Supplementary table 2c. Fatty acid composition (g/100g of total FA) of mature milk (mean HM sampled 60, 90, 120 days after delivery) obtained from women in 7 European countries (mean ± standard deviation (number of observations).**

|  | **ESP** | **FRA** | **ITA** | **NOR** | **PRT** | **ROU** | **SWE** | **All countries** |
| --- | --- | --- | --- | --- | --- | --- | --- | --- |
| Fatty acids (g/100g) | **Mature** | **Mature** | **Mature** | **Mature** | **Mature** | **Mature** | **Mature** | **Mature** |
| MATURE | Mean±std (N) | Mean±std (N) | Mean±std (N) | Mean±std (N) | Mean±std (N) | Mean±std (N) | Mean±std (N) | Mean±std (N) |
| **SFAs** |  |  |  |  |  |  |  |  |
| 6:0 | 0.07±0.01 (7) | 0.08±0.01 (58) | 0.06±0.01 (6) | 0.06±0.01 (9) | 0.06±0.01 (50) | 0.07±0.01 (26) | 0.07±0.02 (28) | 0.07±0.02 (184) |
| 8:0 | 0.20±0.04 (9) | 0.24±0.05 (78) | 0.22±0.05 (14) | 0.21±0.03 (9) | 0.20±0.04 (77) | 0.23±0.05 (35) | 0.20±0.05 (36) | 0.22±0.05 (258) |
| 10:0 | 1.44±0.29 (9) | 1.53±0.30 (85) | 1.55±0.36 (14) | 1.38±0.16 (9) | 1.43±0.30 (79) | 1.49±0.36 (35) | 1.40±0.29 (36) | 1.47±0.31 (267) |
| 12:0 | 5.03±1.72 (10) | 4.97±1.36 (85) | 5.48±1.88 (14) | 5.02±1.17 (9) | 5.43±1.53 (79) | 6.04±1.67 (35) | 5.06±1.30 (36) | 5.29±1.51 (268) |
| 14:0 | 5.03±1.21 (10) | 6.12±1.16 (85) | 5.94±1.74 (14) | 5.49±0.98 (9) | 5.33±1.49 (79) | 5.99±1.71 (35) | 6.36±1.61 (36) | 5.83±1.47 (268) |
| 16:0 | 19.35±2.50(10) | 23.25±1.83 (85) | 22.48±2.47 (14) | 20.09±2.21 (9) | 21.75±1.83 (79) | 21.82±2.25 (35) | 22.03±2.62 (36) | 22.16±2.26 (268) |
| 18:0 | 5.98±0.69 (10) | 7.54±1.18 (85) | 6.33±1.12 (14) | 6.69±1.30 (9) | 5.59±0.71 (79) | 6.02±0.81 (35) | 6.95±1.21 (36) | 6.54±1.28 (268) |
| 20:0 | 0.19±0.04 (10) | 0.20±0.03 (85) | 0.17±0.02 (14) | 0.19±0.03 (9) | 0.15±0.02 (76) | 0.17±0.03 (35) | 0.18±0.02 (36) | 0.18±0.03 (265) |
| 24:0 | 0.12±0.09 (8) | 0.08±0.0 2 (69) | 0.09±0.03 (6) | 0.07±0.02 (9) | 0.07±0.02 (55) | 0.08±0.03 (28) | 0.06±0.01 (26) | 0.08±0.03 (201) |
| **MUFAs** |  |  |  |  |  |  |  |  |
| 16:1 n-7 | 1.53±0.26 (10) | 2.47±0.56 (85) | 2.02±0.44 (14) | 2.53±0.26 (9) | 2.32±0.59 (79) | 2.07±0.49 (35) | 2.53±0.62 (36) | 2.32±0.59 (268) |
| 18:1 n-7 | 2.29±0.44 (10) | 2.16±0.30 (85) | 2.60±0.31 (14) | 2.62±0.35 (9) | 2.62±0.38 (79) | 2.06±0.34 (35) | 2.82±0.48 (36) | 2.41±0.45 (268) |
| 18:1 n-9 | 38.61±4.02(10) | 37.15±2.77 (85) | 37.19±3.93 (14) | 38.70±2.20 (9) | 34.84±3.32 (79) | 31.31±2.04 (35) | 37.38±3.08 (36) | 35.84±3.65 (268) |
| *trans*-18:1 | 11.40±5.76 (10) | 22.09±6.36 (85) | 14.06±4.98 (14) | 27.97±10.90 (9) | 12.85±6.23 (79) | 21.21±12.02 (35) | 25.31±9.48 (36) | 19.06±9.30 (268) |
| 20:1 n-9 | 0.44±0.19 (10) | 0.44±0.07 (85) | 0.36±0.06 (14) | 0.57±0.11 (9) | 0.36±0.06 (79) | 0.34±0.06 (35) | 0.51±0.11 (36) | 0.41±0.10 (268) |
| 22:1 n-9 | 0.11±0.07 (7) | 0.09±0.04 (73) | 0.08±0.03 (7) | 0.10±0.02 (9) | 0.06±0.02 (51) | 0.07±0.02 (26) | 0.09±0.02 (36) | 0.08±0.03 (209) |
| 24:1 n-9 | 0.15±0.17 (6) | 0.08±0.02 (69) | 0.07±0.02 (7) | 0.08±0.02 (9) | 0.07±0.02 (47) | 0.07±0.03 (23) | 0.07±0.01 (33) | 0.08±0.04 (194) |
| **PUFAs** |  |  |  |  |  |  |  |  |
| **18:2 n-6** | **16.69±1.43(10)** | **10.51±1.46 (85)** | **12.71±3.18 (14)** | **12.05±1.77 (9)** | **16.69±3.41 (79)** | **19.10±4.24 (35)** | **10.57±2.24 (36)** | **13.86±4.38 (268)** |
| 20:2 n-6 | 0.46±0.46 (10) | 0.24±0.04 (85) | 0.27±0.06 (14) | 0.26±0.04 (9) | 0.34±0.06 (79) | 0.40±0.08 (35) | 0.23±0.04 (36) | 0.30±0.12 (268) |
| **18:3 n-3** | **0.59±0.24 (10)** | **0.93±0.26 (85)** | **0.52±0.13 (14)** | **1.80±0.49 (9)** | **0.69±0.20 (79)** | **0.53±0.17 (35)** | **1.61±0.54 (36)** | **0.89±0.47 (268)** |
| 18:3 n-6 | 0.16±0.03 (9) | 0.11±0.03 (74) | 0.13±0.03 (12) | 0.09±0.02 (9) | 0.14±0.0 5(75) | 0.14±0.04 (34) | 0.09±0.03 (33) | 0.12±0.04 (246) |
| 20:3 n-6 | 0.48±0.22 (10) | 0.34±0.0 7(85) | 0.36±0.11 (14) | 0.28±0.05 (9) | 0.43±0.10 (79) | 0.44±0.12 (35) | 0.27±0.04 (36) | 0.38±0.11 (268) |
| **20:4 n-6** | **0.48±0.16 (10)** | **0.43±0.07 (85)** | **0.41±0.07 (14)** | **0.38±0.02 (9)** | **0.52±0.0 9(79)** | **0.57±0.11 (35)** | **0.35±0.06 (36)** | **0.46±0.11 (268)** |
| 20:5 n-3 | 0.11±0.07 (4) | 0.09±0.03 (72) | 0.15±0.13 (10) | 0.18±0.10 (9) | 0.10±0.05 (52) | 0.10±0.05 (16) | 0.13±0.05 (34) | 0.11±0.05 (197) |
| **22:6 n-3** | **0.35±0.10 (10)** | **0.33±0.11 (85)** | **0.39±0.23 (14)** | **0.52±0.21 (9)** | **0.40±0.18 (79)** | **0.26±0.09 (35)** | **0.37±0.16 (36)** | **0.36±0.16 (268)** |
| ARA/DHA | 1.52±0.65 (10) | 1.52±0.59 (85) | 1.67±1.28 (14) | 0.91±0.37 (9) | 1.57±0.68 (79) | 2.55±1.00 (35) | 1.18±0.49 (36) | 1.60±0.80 (268) |
| Total SFAs | 37.11 ± 4.33 (10) | 43.96 ± 4.74 (85) | 42.28 ± 5.66 (14) | 39 ± 5.81 (9) | 40.04 ± 4.48 (79) | 41.69 ± 5.55 (35) | 42.28 ± 6.19 (36) | 37.11 ± 4.33 (268) |
| Total MUFAs | 43.91 ± 4.51 (10) | 43.1 ± 3.79 (85) | 43.39 ± 5.11 (14) | 45.31 ± 3.29 (9) | 40.7 ± 4.35 (79) | 36.81 ± 3.15 (35) | 44.12 ± 3.98 (36) | 43.91 ± 4.51 (268) |
| Total PUFAs | 18.98 ± 3.32 (10) | 12.95 ± 2.6 (85) | 14.33 ± 3.49 (14) | 15.69 ± 3.26 (9) | 19.26 ± 4.48 (79) | 21.51 ± 5.43 (35) | 13.6 ± 3.32 (36) | 18.98 ± 3.32(268) |

**Supplementary table 3a. Fatty acid concentration (mg/100 ml) of 7 European countries in colostrum (0±3 days after delivery). Results are expressed as mean ± standard deviation (std) with in brackets the n of observations.**

|  | **ESP** | **FRA** | **ITA** | **NOR** | **PRT** | **ROU** | **SWE** |
| --- | --- | --- | --- | --- | --- | --- | --- |
| Fatty acids  (mg/100 mL) | **Colostrum** | **Colostrum** | **Colostrum** | **Colostrum** | **Colostrum** | **Colostrum** | **Colostrum** |
| COLOSTRUM | Mean±std (N) | Mean±std (N) | Mean±std (N) | Mean±std (N) | Mean±std (N) | **Mean±std (N)** | Mean±std (N) |
| **SFAs** |  |  |  |  |  |  |  |
| 6:0 | 2.24 (1) | 3.25 (1) | - | - | - | - | - |
| 8:0 | 4.87±1.75 (2) | 5.27±6.45 (6) | - | 2.14 (1) | 5.45±2.03 (4) | 7.29±3.73 (6) | 3.59±0.94 (3) |
| 10:0 | 13.83±12.97 (7) | 10.87±14.3 8(79) | 7.69±5.70 (8) | 7.89±7.29 (7) | 7.31±10.12 (70) | 16.04±22.85 (29) | 9.22±7.69 (32) |
| 12:0 | 59.69±54.44 (10) | 59.42±54.79 (80) | 46.32±32.58 (8) | 48.86±40.82 (9) | 40.31±39.42 (87) | 65.11±86.76 (38) | 55.00±37.29 (37) |
| 14:0 | 103.31±91.24 (10) | 117.65±65.94 (80) | 96.48±57.05 (8) | 111.33±79.00 (9) | 88.97±57.27 (87) | 96.61±86.70 (38) | 127.19±66.42 (37) |
| 16:0 | 431.95±217.22(10) | 468.31±213.19(80) | 482.54±267.23 (8) | 469.09±381.80 (9 | 487.21±275.17 (87) | 473.61±259.70 (38) | 520.13±263.67 (37) |
| 18:0 | 114.59±66.52 (10) | 120.35±64.46 (80) | 100.46±52.65(8) | 141.30±93.06 (9) | 104.41±58.36 (87) | 111.18±58.30 (38) | 141.07±76.22 (37) |
| 20:0 | 4.60±2.44 (9) | 4.52±2.13 (78) | 3.99±0.90 (6) | 5.56±2.69 (9) | 4.17±1.91 (76) | 4.09±1.70 (34) | 5.56±2.34 (34) |
| 24:0 | 3.97±1.83 (10) | 4.09±1.74 (78) | 3.74±0.99 (6) | 4.98±1.60 (9) | 4.43±2.10 (78) | 4.02±1.67 (36) | 4.94±2.07 (34) |
| **MUFAs** |  |  |  |  |  |  |  |
| 16:1 n-7 | 30.15±19.21 (10) | 40.09±18.63 (80) | 48.38±33.44 (8) | 38.61-33.77 (9) | 39.87±27.18 (87) | 38.49±27.08(38) | 40.69±26.79 (37) |
| 18:1 n-7 | 57.43±27.72 (10) | 50.52±21.15 (80) | 75.17±44.57 (8) | 61.29±43.86 (9) | 63.74±37.71 (87) | 49.56±26.72(38) | 69.20±36.89 (37) |
| 18:1 n-9 | 818.17±373.82(10) | 682.83±300.11(80) | 728.52±394.45 (8) | 658.18±407.75(9) | 659.93±371.79 (87) | 642.66±336.71(38) | 743.28±382.99 (37) |
| 20:1 n-9 | 16.40±6.23 (10) | 16.75±7.84 (80) | 15.90±7.45 (8) | 19.43±11.11 (9) | 15.52±9.06 (87) | 13.67±6.90(38) | 21.16±10.94 (36) |
| 22:1 n-9 | 4.10±1.65 (10) | 4.21±1.7 3(74) | 4.34±1.33 (7) | 5.30±2.28 (8) | 4.49±2.20 (72) | 3.84±1.55(30) | 5.29±2.61 (35) |
| 24:1 n-9 | 6.89±2.87 (10) | 6.54±3.01 (80) | 7.30±2.30 (7) | 8.35±4.04 (9) | 7.32±3.9 1(86) | 5.96±2.63(38) | 8.50±4.31 (36) |
| **PUFAs** |  |  |  |  |  |  |  |
| 18:2 n-6 | 309.31±170.13(10) | 183.75±86.64 (80) | 238.22±144.84 (8) | 180.90±118.01(9) | 264.70±154.25 (87) | 315.02±180.56 (38) | 182.46±114.24 (37) |
| 20:2 n-6 | 16.74±9.50 (10) | 11.69±5.81 (79) | 16.10±6.58 (7) | 12.78±7.27 (9) | 17.40±10.24 (87) | 18.87±9.72 (38) | 12.89±7.29 (36) |
| 18:3 n-3 | 9.47±5.95 (10) | 19.27±10.69 (80) | 13.51±10.37 (8) | 20.83±13.15 (9) | 11.36±7.17 (85) | 8.73±5.6 1(36) | 25.93±15.0 1(36) |
| 18:3 n-6 | 3.70±1.4 0(2) | 3.22±1.42 (3) | 3.20±0.45 (3) | - | 3.20±1.54 (6) | 3.79±1.95 (6) | - |
| 20:3 n-6 | 11.73±5.33 (10) | 10.97±4.95 (80) | 17.34±11.31 (7) | 10.78±5.84 (9) | 15.67±10.23 (87) | 14.04±7.81 (38) | 11.65±6.56 (36) |
| 20:4 n-6 | 13.35±5.15 (10) | 15.15±6.7 2(80) | 15.27±8.65 (8) | 13.85±7.10 (9) | 18.83±11.44 (87) | 17.52±8.13 (38) | 14.06±8.0 9(37) |
| 20:5 n-3 | 3.01 (1) | 2.73±0.90 (18) | - | 4.99±2.06 (4) | 2.91±1.24 (12) | 7.87 (1) | 2.95±1.18 (8) |
| 22:6 n-3 | 9.60±4.81 (10) | 12.96±6.48 (80) | 13.47±7.13 (7) | 19.92±14.96 (9) | 13.59±9.06 (87) | 8.04±6.03 (36) | 14.47±9.86 (37) |
| ARA/DHA | 1.53±0.41 (10) | 1.26±0.34 (80) | 1.55±0.60 (8) | 0.92±0.44 (9) | 1.49±0.44 (87) | 2.73±1.13 (38) | 1.06±0.52 (37) |
| Total SFAs (g/100mL) | 0.73±0.42 (10) | 0.78±0.40 (80) | 0.74±0.41 (8) | 0.79±0.52 (9) | 0.74±0.42 (87) | 0.77±0.48 (38) | 0.86±0.44 (37) |
| Total MUFAs (g/100mL) | 0.94±0.43 (10) | 0.81±0.35 (80) | 0.89±0.49 (8) | 0.80±0.51 (9) | 0.80±0.45 (87) | 0.77±0.40 (38) | 0.90±0.46 (37) |
| Total PUFAs (g/100mL) | 0.37±0.19 (10) | 0.26±0.12 (80) | 0.31±0.19 (8) | 0.26±0.16 (9) | 0.34±0.20 (87) | 0.38±0.21 (38) | 0.26±0.15 (37) |
|  |  |  |  |  |  |  |  |

**Supplementary table 3b. Fatty acid concentration (mg/100 ml) of 7 European countries in transitional milk (17±5 days after delivery). Results are expressed as mean ± standard deviation (std) with in brackets the n of observations.**

|  | **ESP** | **FRA** | **ITA** | **NOR** | **PRT** | | **ROU** | | **SWE** | |  |
| --- | --- | --- | --- | --- | --- | --- | --- | --- | --- | --- | --- |
| Fatty acids  (mg/100 ml) | **Transitional** | **Transitional** | **Transitional** | **Transitional** |  | **Transitional** | | **Transitional** | | **Transitional** | |
|  |  |  |  |  |  |  | |  | |  | |
| TRANSITIONAL | Mean±std (N) | Mean±std (N) | Mean±std (N) | Mean±std (N) |  | Mean±std (N) | | Mean±std (N) | | Mean±std (N) | |
| **SFAs** |  |  |  |  |  |  | |  | |  | |
| 6:0 | - | 2.44±0.34 (10) | - | 2.75 (1) |  | 2.42±0.44 (4) | | 2.33±0.27 (8) | | 2.64±0.50 (3) | |
| 8:0 | 5.70±1.49 (8) | 7.76±3.12 (50) | 5.56±1.82 (12) | 7.17±2.49 (10) |  | 5.87±2.46 (94) | | 6.70±3.32 (39) | | 7.48±2.74 (40) | |
| 10:0 | 39.21±15.46 (9) | 50.59±19.99 (84) | 38.88±14.90 (13) | 49.99±16.71 (10) |  | 45.16±19.83 (95) | | 46.47±23.52 (40) | | 53.53±22.74 (41) | |
| 12:0 | 152.05±59.28 (9) | 169.88±81.43 (84) | 141.61±61.05 (13) | 175.19±65.07 (10) |  | 174.56±88.06 (95) | | 181.26±92.87 (40) | | 193.20±95.16 (41) | |
| 14:0 | 140.03±55.49 (9) | 199.90±87.52 (84) | 155.23±63.46 (13) | 185.81±68.88 (10) |  | 169.04±87.04 (95) | | 178.03±88.20 (40) | | 228.07±108.89 (41) | |
| 16:0 | 493.22±147.35 (9) | 696.44±259.57 (84) | 572.65±285.55 (13) | 708.07±231.20 (10) |  | 636.66±272.16 (95) | | 643.22±286.64 (40) | | 760.22±296.37 (41) | |
| 18:0 | 147.98±40.58 (9) | 218.81±91.98 (84) | 151.46±87.45 (13) | 228.63±93.75 (10) |  | 150.87±68.52 (95) | | 159.93±68.97 (40) | | 234.36±105.51 (41) | |
| 20:0 | 4.79±1.49 (9) | 6.53±2.53 (82) | 4.65±1.94 (11) | 7.09±2.99 (10) |  | 4.72±2.02 (88) | | 4.90±1.93 (37) | | 6.66±3.23 (40) | |
| 24:0 | 3.75±1.55 (7) | 3.36±0.93 (66) | 2.55±0.31 (7) | 3.03±1.01 (10) |  | 3.18±0.91 (59) | | 3.48±1.19 (27) | | 3.40±1.24 (27) | |
| **MUFAs** |  |  |  |  |  |  | |  | |  | |
| 16:1 n-7 | 36.89±16.28 (9) | 71.62±30.39 (84) | 59.29±34.10 (13) | 91.29±46.50 (10) |  | 71.11±39.47 (95) | | 62.32±32.65 (40) | | 83.00±36.10 (41) | |
| 18:1 n-7 | 61.95±18.90 (9) | 64.72±24.91 (84) | 70.08±33.36 (13) | 90.40±35.45 (10) |  | 77.20±34.32 (95) | | 65.92±33.27 (40) | | 100.26±44.05 (41) | |
| 18:1 n-9 | 1016.12±380.33 (9) | 1062.06±374.83 (84) | 897.49±418.94 (13) | 1238.70±385.77 (10) |  | 963.44±445.40 (95) | | 869.46±403.12 (40) | | 1286.14±577.81 (41) | |
| 20:1 n-9 | 13.42±4.56 (9) | 15.54±6.87 (84) | 11.93±7.06 (13) | 19.67±6.43 (10) |  | 12.23±6.10 (95) | | 11.08±5.23 (40) | | 20.08±9.79 (41) | |
| 22:1 n-9 | 3.35±0.87 (7) | 3.57±1.31 (64) | 3.09±1.10 (6) | 3.77±1.12 (9) |  | 3.18±1.05 (54) | | 3.04±1.12 (23) | | 4.27±1.77 (34) | |
| 24:1 n-9 | 4.07±2.36 (7) | 3.56±1.25 (65) | 2.87±0.69 (8) | 3.49±0.89 (9) |  | 3.51±1.39 (63) | | 3.43±1.21 (30) | | 4.09±1.47 (33) | |
| **PUFAs** |  |  |  |  |  |  | |  | |  | |
| 18:2 n-6 | 416.45±187.82 (9) | 303.21±125.72 (84) | 286.52±133.44 (13) | 390.69±93.68 (10) |  | 443.13±214.53 (95) | | 506.15±266.76 (40) | | 364.21±200.16 (41) | |
| 20:2 n-6 | 13.04±5.27 (9) | 9.28±3.99 (84) | 8.00±3.55 (13) | 11.10±3.37 (10) |  | 12.69±6.11 (95) | | 14.68±6.94 (40) | | 10.49±5.83 (41) | |
| 18:3 n-3 | 19.09±17.39 (9) | 27.76±13.66 (84) | 14.71±8.90 (13) | 46.10±19.96 (10) |  | 18.62±11.57 (95) | | 14.33±8.24 (40) | | 54.05±33.63 (41) | |
| 18:3 n-6 | 4.44±1.25 (7) | 3.20±1.18 (47) | 3.77±1.93 (7) | 3.67±1.61 (8) |  | 4.57±2.91 (69) | | 4.41±2.45 (30) | | 3.42±1.20 (29) | |
| 20:3 n-6 | 16.00±5.15 (9) | 12.00±4.78 (84) | 11.02±6.27 (13) | 13.37±4.80 (10) |  | 16.54±8.94 (95) | | 16.12±9.36 (40) | | 12.86±5.33 (41) | |
| 20:4 n-6 | 13.82±4.12 (9) | 15.73±5.66 (84) | 12.65±5.93 (13) | 16.43±4.72 (10) |  | 18.48±8.42 (95) | | 19.51±8.65 (40) | | 15.30±6.84 (41) | |
| 20:5 n-3 | 4.64 (1) | 3.79±2.39 (45) | 4.60±1.59 (4) | 6.32±2.93 (9) |  | 3.57±1.80 (30) | | 2.75±0.72 (9) | | 4.46±2.13 (33) | |
| 22:6 n-3 | 11.39±6.12 (9) | 14.87±11.65 (84) | 10.67±7.38 (13) | 21.15±10.10 (10) |  | 14.69±9.39 (95) | | 9.06±5.33 (39) | | 16.42±9.35 (41) | |
| ARA/DHA | 1.38±0.45 (9) | 1.26±0.42 (84) | 1.52±0.80 (13) | 0.87±0.29 (10) |  | 1.45±0.55 (95) | | 2.56±1.06 (40) | | 1.04±0.32 (41) | |
| Total SFAs (g/100ml) | 0.99±0.29 (9) | 1.35±0.50 (84) | 1.07±0.47 (13) | 1.37±0.42 (10) |  | 1.19±0.50 (95) | | 1.22±0.51 (40) | | 1.49±0.58 (41) | |
| Total MUFAs (g/100ml) | 1.15±0.41 (9) | 1.24±0.44 (84) | 1.06±0.50 (13) | 1.47±0.47 (10) |  | 1.14±0.52 (95) | | 1.03±0.48 (40) | | 1.52±0.66 (41) | |
| Total PUFAs (g/100ml) | 0.49±0.21 (9) | 0.39±0.15 (84) | 0.35±0.16 (13) | 0.51±0.12 (10) |  | 0.53±0.25 (95) | | 0.58±0.30 (40) | | 0.48±0.25 (41) | |
|  |  |  |  |  |  |  | |  | |  | |

**Supplementary table 3c. Fatty acid concentration (mg/100 ml) of 7 European countries in mature milk (mean HM sampled 60, 90, 120 days after delivery). Results are expressed as mean ± standard deviation (std) with in brackets the n of observations.**

|  | **ESP** | **FRA** | **ITA** | **NOR** | **PRT** | **ROU** | **SWE** | **All countries** |
| --- | --- | --- | --- | --- | --- | --- | --- | --- |
| Fatty acids  (mg/100 ml) | **Mature** | **Mature** | **Mature** | **Mature** | **Mature** | **Mature** | **Mature** | **Mature** |
| MATURE | Mean±std (N) | Mean±std (N) | Mean±std (N) | Mean±std (N) | Mean±std (N) | Mean±std (N) | Mean±std (N) | Mean±std (N) |
| SFAs |  |  |  |  |  |  |  |  |
| 6:0 | 2,91±0.51 (7) | 2.89±0.79 (58) | 2.79±0.45 (6) | 2.86±0.51 (9) | 2.83±0.66 (50) | 2.61±0.57 (26) | 2.87±1.11 (28) | 2.83±0.76 (184) |
| 8:0 | 6.26±2.34 (9) | 7.22±2.5 7(78) | 5.65±1.84 (14) | 8.72±1.37 (9) | 5.91±2.17 (77) | 6.94±2.3 8(35) | 7.13±2.18 (36) | 6.71±2.38 (258) |
| 10:0 | 41.90±15.94 (9) | 44.73±15.4 5(85) | 38.96±13.67 (14) | 57.54±8.74 (9) | 39.70±16.15 (79) | 44.88±16.38 (35) | 48.35±16.51 (36) | 43.78±16.03 (267) |
| 12:0 | 149.17±86.63 (10) | 147.48±64.9 8(85) | 136.37±53.59 (14) | 208.09±36.22 (9) | 150.06±66.58 (79) | 183.75±89.28 (35) | 177.17±69.15 (36) | 158.48±70.01 (268) |
| 14:0 | 144.40±82.95 (10) | 180.54±64.56 (85) | 146.36±48.86 (14) | 228.47±42.12 (9) | 147.77±67.27 (79) | 180.23±79.16 (35) | 222.33±83.45 (36) | 174.93±73.86 (268) |
| 16:0 | 526.01±249.94 (10) | 678.80±169.9 9(85) | 573.39±208.09 (14) | 840.10±186.39 (9) | 615.54±245.20 (79) | 671.51±240.85 (35) | 773.03±242.58 (36) | 666.07±227.09(268) |
| 18:0 | 160.54±69.62 (10) | 220.18±61.18 (85) | 162.62±66.59 (14) | 276.81±66.51 (9) | 157.55±61.17 (79) | 183.86±66.45 (35) | 242.43±74.56 (36) | 196.63±73.02 (268) |
| 20:0 | 5.04±1.76 (10) | 6.00±1.6 5(85) | 4.57±1.46 (14) | 7.99±1.19 (9) | 4.86±1.75 (76) | 5.31±1.77 (35) | 6.38±1.73 (36) | 5.59±1.83 (265) |
| 24:0 | 2.99±0.68 (8) | 2.89±0.70 (69) | 2.82±0.40 (6) | 2.95±0.50 (9) | 3.02±0.71 (55) | 3.25±1.07 (28) | 2.97±0.66 (36) | 2.99±0.75 (201) |
| **MUFAs** |  |  |  |  |  |  |  |  |
| 16:1 n-7 | 42.34±20.94 (10) | 71.12±21.49 (85) | 52.02±24.15 (14) | 107.21±27.45 (9) | 66.64±32.42 (79) | 64.05±25.39 (35) | 88.52±33.95 (36) | 70.35±29.97 (268) |
| 18:1 n-7 | 60.19±28.39 (10) | 63.48±19.91 (85) | 67.16±27.11 (14) | 112.04±33.04 (9) | 72.82±28.15 (79) | 62.55±21.04 (35) | 98.66±32.86 (36) | 72.54±29.00 (268) |
| 18:1 n-9 | 1074.23±468.69 (10) | 1090.61±298.00 (85) | 971.63±407.08 (14) | 1639.82±355.92 (9) | 989.20±405.54 (79) | 964.54±329.21 (35) | 1320.49±433.75 (36) | 1086.75±396.07 (268) |
| 20:1 n-9 | 11.12±3.82 (10) | 13.13±4.45 (85) | 9.50±3.74 (14) | 24.50±7.07 (9) | 10.37±4.23 (79) | 10.52±3.82 (35) | 18.20±7.16 (36) | 12.77±5.87 (268) |
| 22:1 n-9 | 3.09±1.24 (7) | 3.29±1.29 (73) | 2.88±0.68 (7) | 4.34±1.18 (9) | 2.84±0.67 (51) | 2.87±0.69 (26) | 3.57±1.19 (36) | 3.20±1.10 (209) |
| 24:1 n-9 | 3.29±0.68 (6) | 2.83±0.78 (69) | 2.53±0.40 (7) | 3.57±0.77 (9) | 2.81±0.64 (47) | 3.04±1.02 (23) | 3.12±0.83 (33) | 2.94±0.79 (194) |
| **PUFAs** |  |  |  |  |  |  |  |  |
| **18:2 n-6** | **466.97±223.31 (10)** | **314.02±108.80 (85)** | **326.46±139.47 (14)** | **515.79±150.11 (9)** | **476.91±238.42 (79)** | **584.69±229.05 (35)** | **375.24±135.72 (36)** | **418.74±204.79 (268)** |
| 20:2 n-6 | 10.21±4.23 (10) | 7.33±2.51 (85) | 6.88±2.77 (14) | 11.08±2.97 (9) | 9.67±4.04 (79) | 12.37±4.3 1(35) | 8.43±2.92 (36) | 9.04±3.81 (268) |
| **18:3 n-3** | **16.76±9.82 (10)** | **27.62±12.52 (85)** | **12.99±4.73 (14)** | **79.04±30.15 (9)** | **19.60±10.82 (79)** | **15.63±6.48 (35)** | **57.32±27.00 (36)** | **28.24±21.84 (268)** |
| 18:3 n-6 | 5.12±1.46 (9) | 3.74±1.0 9 (74) | 3.81±0.81 (12) | 4.12±1.22 (9) | 4.62±2.08 (75) | 4.63±1.74 (34) | 3.60±1.17 (33) | 4.18±1.62 (246) |
| 20:3 n-6 | 11.68±4.12 (10) | 9.96±3.18 (85) | 9.11±3.83 (14) | 11.84±2.87 (9) | 12.49±5.65 (79) | 13.28±4.57 (35) | 9.64±3.19 (36) | 11.18±4.49 (268) |
| **20:4 n-6** | **12.41±5.47 (10)** | **12.66±3.81 (85)** | **10.41±3.35 (14)** | **16.12±3.52 (9)** | **14.89±6.64 (79)** | **17.34±5.75 (35)** | **12.47±4.47 (36)** | **13.89±5.45 (268)** |
| 20:5 n-3 | 4.03±1.11 (4) | 3.15±1.17 (72) | 3.85±1.72 (10) | 7.24±4.00 (9) | 3.53±1.81 (52) | 3.43±1.69 (16) | 4.85±2.06 (34) | 3.80±2.00 (197) |
| **22:6 n-3** | **9.26±3.64 (10)** | **9.77±4.68 (85)** | **9.90±4.60 (14)** | **21.25±8.74 (9)** | **11.48±7.57 (79)** | **7.73±2.91 (35)** | **13.60±7.83 (36)** | **10.90±6.56 (268)** |
| ARA/DHA | 1.54±0.55 (10) | 1.51±0.47 (85) | 1.71±1.2 2(14) | 0.88±0.34 (9) | 1.57±0.58 (79) | 2.60±0.85 (35) | 1.16±0.44 (36) | 1.61±0.74 (268) |
| Total SFAs (g/100ml) | 1.03±0.50 (10) | 1.29±0.35 (85) | 1.07±0.35 (14) | 1.63±0.28 (9) | 1.12±0.43 (79) | 1.28±0.46 (35) | 1.48±0.45 (36) | 1.25±0.43 (268) |
| Total MUFAs (g/100ml) | 1.20±0.52 (10) | 1.26±0.34 (85) | 1.12±0.46 (14) | 1.92±0.43 (9) | 1.15±0.47 (79) | 1.13±0.38 (35) | 1.56±0.50 (36) | 1.27±0.46 (268) |
| Total PUFAs (g/100ml) | 0.53±0.25 (10) | 0.39±0.13 (85) | 0.38±0.15 (14) | 0.67±0.19 (9) | 0.55±0.27 (79) | 0.66±0.24 (35) | 0.48±0.18 (36) | 0.50±0.23 (268) |
|  |  |  |  |  |  |  |  |  |

**Supplementary table 4. Correlation (rho/p) between fatty acid composition in human milk at different lactation stages (most abundant milk FAs and most physiologically relevant PUFAs were considered) and mother plasma, erythrocytes and adipose tissue at delivery (Spearman correlation). Bold letters are correlations considered to be relevant and significant because rho>0.4 and p<0.05.**

| **Fatty acids** | | **Plasma (N=264)** | | | **Erythrocytes (N=264)** | | | **Adipose Tissue (N=38)** | | |
| --- | --- | --- | --- | --- | --- | --- | --- | --- | --- | --- |
| **Plasma, Erythrocytes or Adipose Tissue** | **Human milk** | **Colostrum** | **Transitional milk** | **Mature* milk** | **Colostrum** | **Transitional milk** | **Mature milk** | **Colostrum** | **Transitional milk** | **Mature milk** |
| 12:0 | 12:0 | 0.16/0.02 | -0.15/0.02 | -0.15/0.03 | - | - | - | -0.09/0.63 | 0.19/0.28 | 0.28/0.15 |
| 14:0 | 14:0 | **0.42/0.001** | 0.02/0.77 | 0.07/0.32 | 0.20/0.00 | 0.04/0.54 | 0.12/0.08 | 0.29/0.11 | 0.35/0.04 | 0.35/0.06 |
| 16:0 | 16:0 | 0.32/0.001 | 0.11/0.11 | 0.17/0.01 | 0.17/0.01 | 0.06/0.37 | -0.08/0.24 | **0.61/0.001** | -0.02/0.91 | **0.64/0.001** |
| 18:0 | 18:0 | 0.16/0.02 | 0.09/0.16 | -0.07/0.30 | 0.09/0.21 | -0.10/0.12 | -0.17/0.01 | **0.47/0.01** | 0.07/0.68 | **0.53/0.001** |
| **18:1 n-9** | **18:1 n-9** | **0.53/0.001** | **0.43/0.001** | **0.51/0.001** | 0.12/0.09 | 0.16/0.02 | 0.13/0.06 | **0.44/0.01** | 0.00/0.99 | 0.08/0.69 |
| **18:2 n-6** | **18:2 n-6** | **0.67/0.001** | **0.55/0.001** | **0.63/0.001** | -0.01/0.86 | -0.08/0.24 | -0.02/0.74 | **0.64/0.001** | **0.42/0.01** | **0.65/0.001** |
| **18:3 n-3** | **18:3 n-3** | **0.77/0.001** | **0.67/0.001** | **0.71/0.001** | **0.55/0.001** | **0.44/0.001** | **0.41/0.001** | **0.72/0.001** | 0.33/0.05 | **0.82/0.001** |
| **20:4 n-6** | **20:4 n-6** | **0.37/0.001** | **0.56/0.001** | **0.56/0.001** | -0.18/0.13 | -0.15/0.18 | -0.21/0.08 | **0.45/0.01** | 0.28/0.11 | **0.60/0.001** |
| **20:5 n-3** | **20:5 n-3** | **0.45/0.01** | **0.46/0.001** | 0.34/0.001 | 0.28/0.13 | 0.36/0.001 | 0.21/0.01 | **0.40/0.75** | 0.33/0.43 | -0.25/0.45 |
| **22:6 n-3** | **22:6 n-3** | **0.47/0.001** | **0.61/0.001** | **0.55/0.001** | 0.13/0.07 | 0.25/0.001 | 0.19/0.01 | **0.79/0.001** | **0.77/0.001** | **0.65/0.001** |
| 18:2 n-6 | 20:4 n-6 | -0.00/0.97 | -0.09/0.17 | -0.09/0.02 | -0.09/0.20 | -0.25/0.001 | -0.15/0.001 | -0.31/0.08 | 0.20/0.25 | 0.06/0.54 |
| 18:3 n-3 | 20:5 n-3 | 0.22/0.24 | 0.17/0.08 | 0.28/0.001 | 0.22/0.001 | 0.18/0.01 | 0.15/0.03 | -0.2/0.92 | 0.27/0.42 | -0.3/0.25 |
| **18:3 n-3** | **22:6 n-3** | 0.21/0.001 | 0.16/0.02 | 0.17/0.01 | 0.22/0.24 | 0.17/0.08 | 0.28/0.001 | **0.56/0.001** | **0.58/0.001** | **0.52/0.001** |
| SFAs | SFAs | 0.37/0.001 | 0.06/0.37 | 0.09/0.18 | 0.06/0.43 | 0.08/0.29 | 0.04/0.65 | 0.26/0.23 | 0.25/0.26 | 0.15/0.24 |
| **MUFAs** | **MUFAs** | **0.50/0.001** | **0.40/0.001** | **0.52/0.001** | 0.02/0.75 | -0.04/0.61 | -0.01/0.90 | **0.45/0.01** | 0.04/0.81 | 0.29/0.001 |
| **PUFAs** | **PUFAs** | **0.59/0.001** | **0.48/0.001** | **0.60/0.001** | -0.21/0.01 | -0.17/0.02 | -0.16/0.04 | **0.54/0.02** | **0.55/0.01** | **0.54/0.001** |

## *from 30 to 120 days.

**Supplementary table 5. Correlation (rho/p) between fatty acid composition in maternal and cord blood plasma and erythrocytes at birth (Spearman correlation). Bold letters are correlations considered to be relevant and significant because rho>0.4 and p<0.05.**

| **Maternal blood FA** | **Plasma & Erythrocytes Cord blood FA** | **Plasma Cord blood (N=87)**  **rho/p** | **Erythrocytes Cord blood (N=87)**  **rho/p** |
| --- | --- | --- | --- |
| 12:0 | 12:0 | 0.31/0.001 | - |
| 14:0 | 14:0 | 0.30/0.01 | 0.34/0.001 |
| 16:0 | 16:0 | 0.31/0.001 | **0.49/0.001** |
| 18:0 | 18:0 | 0.23/0.04 | 0.37/0.001 |
| 18:1 n-9 | 18:1 n-9 | 0.35/0.001 | 0.27/0.01 |
| 18:2 n-6 | 18:2 n-6 | 0.35/0.001 | **0.41/0.001** |
| **18:3 n-3** | **18:3 n-3** | **0.54/0.001** | 0.08/0.49 |
| **20:4 n-6** | **20:4 n-6** | 0.23/0.03 | **0.51/0.001** |
| **20:5 n-3** | **20:5 n-3** | **0.81/0.001** | **0.61/0.001** |
| **22:6 n-3** | **22:6 n-3** | **0.66/0.001** | **0.54/0.001** |
| **18:2 N-6** | **20:4 N-6** | -0.10/0.36 | 0.35/0.001 |
| **18:3 n-3** | **20:5 n-3** | **0.57/0.001** | 0.31/0.001 |
| **18:3 n-3** | **22:6 n-3** | 0.31/0.001 | 0.23/0.03 |
| SFAs | SFAs | 0.35/0.001 | **0.48/0.001** |
| **MUFAs** | **MUFAs** | **0.42/0.001** | **0.56/0.001** |
| PUFAs | PUFAs | 0.35/0.001 | **0.62/0.001** |

**Supplementary table 6. Number of mothers who had DHA supplements and mean fraction of DHA human milk in not supplemented and supplemented groups.**

|  | **Not Supplemented** | | | **Supplemented** | | |
| --- | --- | --- | --- | --- | --- | --- |
|  | DHA human milk fraction (g/100g) | | | | | |
| Time point | N | Mean | sd | N | Mean | sd |
| (0-3) d | 254 | 0.68 | 0.26 | 12 | 0.85 | 0.42 |
| 17±3 d | 275 | 0.47 | 0.25 | 16 | 0.50 | 0.17 |
| 30±3 d | 243 | 0.40 | 0.22 | 14 | 0.47 | 0.28 |
| 60±5 d | 220 | 0.34 | 0.16 | 13 | 0.3 | 0.18 |
| 90±5 d | 210 | 0.34 | 0.17 | 12 | 0.35 | 0.12 |
| 120±5 d | 201 | 0.34 | 0.35 | 11 | 0.35 | 0.19 |
